# Involvement of TLR2–TLR4, NLRP3, and IL-17 in pain induced by a novel Sprague-Dawley rat model of experimental autoimmune encephalomyelitis

**DOI:** 10.3389/fpain.2022.932530

**Published:** 2022-09-13

**Authors:** Andrew J. Kwilasz, Madison A. Clements, Tracey A. Larson, Kevin M. Harris, Scott T. Litwiler, Brodie J. Woodall, Laurel S. Todd, Anouk E. W. Schrama, Eric H. Mitten, Steven F. Maier, Anne-Marie Van Dam, Kenner C. Rice, Linda R. Watkins

**Affiliations:** ^1^Department of Psychology and Neuroscience, University of Colorado, Boulder, CO, United States; ^2^The Center for Neuroscience, University of Colorado, Boulder, CO, United States; ^3^Department of Anatomy and Neuroscience, Amsterdam UMC, Vrije Universiteit, Amsterdam, Netherlands; ^4^Drug Design and Synthesis Section, National Institute on Drug Abuse and National Institute on Alcohol Abuse and Alcoholism, National Institutes of Health, Bethesda, MD, United States

**Keywords:** Sprague-Dawley rats, TLR2–TLR4, NLRP3, interleukin-17, experimental autoimmune encephalomyelitis

## Abstract

Up to 92% of patients suffering from multiple sclerosis (MS) experience pain, most without adequate treatment, and many report pain long before motor symptoms associated with MS diagnosis. In the most commonly studied rodent model of MS, experimental autoimmune encephalomyelitis (EAE), motor impairments/disabilities caused by EAE can interfere with pain testing. In this study, we characterize a novel low-dose myelin-oligodendrocyte-glycoprotein (MOG)-induced Sprague-Dawley (SD) model of EAE-related pain in male rats, optimized to minimize motor impairments/disabilities. Adult male SD rats were treated with increasing doses of intradermal myelin-oligodendrocyte-glycoprotein (MOG_1−125_) (0, 4, 8, and 16 μg) in incomplete Freund's adjuvant (IFA) vehicle to induce mild EAE. Von Frey testing and motor assessments were conducted prior to EAE induction and then weekly thereafter to assess EAE-induced pain and motor impairment. Results from these studies demonstrated that doses of 8 and 16 μg MOG_1−125_ were sufficient to produce stable mechanical allodynia for up to 1 month in the absence of hindpaw motor impairments/disabilities. In the follow-up studies, these doses of MOG_1−125_, were administered to create allodynia in the absence of confounded motor impairments. Then, 2 weeks later, rats began daily subcutaneous injections of the Toll-like receptor 2 and 4 (TLR2–TLR4) antagonist (+)-naltrexone [(+)-NTX] or saline for an additional 13 days. We found that (+)-NTX also reverses EAE-induced mechanical allodynia in the MOG-induced SD rat model of EAE, supporting parallels between models, but now allowing a protracted timecourse to be examined completely free of motor confounds. Exploring further mechanisms, we demonstrated that both spinal NOD-like receptor protein 3 (NLRP3) and interleukin-17 (IL-17) are necessary for EAE-induced pain, as intrathecal injections of NLRP3 antagonist MCC950 and IL-17 neutralizing antibody both acutely reversed EAE-induced pain. Finally, we show that spinal glial immunoreactivity induced by EAE is reversed by (+)-NTX, and that spinal demyelination correlates with the severity of motor impairments/disabilities. These findings characterize an optimized MOG-induced SD rat model of EAE for the study of pain with minimal motor impairments/disabilities. Finally, these studies support the role of TLR2–TLR4 antagonists as a potential treatment for MS-related pain and other pain and inflammatory-related disorders.

## Introduction

Multiple sclerosis (MS) is a debilitating inflammatory and demyelinating disease of the central nervous system (CNS). While classical symptoms involve motor impairments/disabilities leading to paresis and/or paralysis, other CNS-related symptoms are common in patients with MS, including increased pain, which has been reported in nearly 92% of patients with MS (1). Despite treatments such as fingolimod and dimethyl fumarate that have been developed for MS, these therapies are only partially effective against classical and non-classical symptoms and can produce severe side effects, including increased pain (2–5). It is thus critically important to develop treatments for MS-related pain and to develop better animal models to assess the efficacy of these treatments.

The most commonly used rodent model of MS is experimental autoimmune encephalomyelitis (EAE). In EAE, spinal cord homogenate or an antigen for myelin such as myelin-oligodendrocyte-glycoprotein (MOG) is administered to subjects to induce an autoimmune reaction against CNS myelin. Standard EAE models produce motor impairments/disabilities that ultimately require euthanasia once they become too severe (6). However, in addition to motor impairments/disabilities, EAE also produces a multitude of other symptoms including memory and cognitive deficits (7–11), anxiety and depression (12, 13), decreased social interaction (14), and pain (14–23), all of which are also commonly reported symptoms of patients with MS (1, 24–28). Modeling such non-motor symptoms of MS in rodents, including pain, typically requires strategies to avoid behavioral testing during severe motor impairments/disabilities that can interfere with behavioral responses. In this study, we characterize a new, low-dose MOG-induced EAE model in Sprague-Dawley (SD) rats that produces minimal motor impairments/disabilities and is thus optimized for the study of EAE-induced pain that is stable across time.

Pain in EAE is mediated by ongoing demyelination as well as various proinflammatory cell types and mediators including cytokines released from both glia and T cells. For example, we have shown that spinal interleukin-1β (IL-1β) is necessary for EAE-induced pain in Dark Agouti (DA) rats (16), and others have demonstrated that IL-1β is necessary for EAE-induced pain in mice (20). Ligation of Toll-like receptors 2 and 4 (TLR2–TLR4) by Danger-associated molecular patterns (DAMPs) induces NF-kB activation, which primes the nod-like receptor protein 3 (NLRP3) inflammasome which can then lead to the production of the proinflammatory cytokine IL-1β (29, 30). Importantly, NLPR3 activation has also been shown to be necessary for EAE pain in mice (31). Moreover, the Th17 cell cytokine interleukin-17 (IL-17) has recently been shown to be necessary for EAE-induced pain (32). Finally, it is well-known that CNS axonal damage from demyelination can also contribute to ongoing pain in disorders such as acute transverse myelitis (33), suggesting that demyelination from MS likely contributes to ongoing pain. Studying these mechanisms in patients with MS remains difficult, due to a lack of experimental tools to manipulate these molecules directly in humans. This further highlights the importance of EAE for exploring mechanisms underlying MS-related symptomatology. Collectively, these studies suggest that treatments that inhibit these proinflammatory molecules and/or improve remyelination would be effective for the treatment of EAE and MS-related pain.

Toll-like receptors 2 and 4 antagonists are one promising strategy to treat pain from MS (16), as well as neuropathic pain from both peripheral and central origin (34–37). Toll-like receptors 2 and 4 are commonly expressed on glial cells (29, 38) as well as less commonly on neurons (38, 39) and T cells (40, 41). Importantly, TLR2–TLR4s are upregulated in white matter of patients with MS (42–44) as well as in EAE (42–45). Various studies have demonstrated the ability of TLR2 or TLR4 knockout to modulate EAE disease progression in mice (40, 41, 46–48). However, the results reported have been ambiguous, as no effect, enhancement, and reduction of EAE motor impairments/disabilities have all been observed across these studies. Notably, specific knockout of TLR2 or TLR4 on CD4+ T cells has been shown to reduce EAE motor impairments/disabilities (40, 41), suggesting that TLR2 and TLR4 expressions on specific cell types likely play different roles in EAE disease expression. However, importantly, all of these studies employed knockout of TLR2 or TLR4 prior to EAE induction, which may also block endogenous proinflammatory signaling that is required for a normal inflammatory response to MOG.

In contrast, we have previously shown that daily systemic administration of the non-opioid low-affinity TLR2–TLR4 antagonist, (+)-naltrexone [(+)-NTX] (49–51), reverses pain induced by low-dose EAE in a DA rat model of pain (16) and have furthermore demonstrated that this same treatment returns dorsal lumbar spinal cord glial activation and NLRP3, IL-1β, and IL-17 mRNAs to control levels (16). Here, we study the role of TLR2–TLR4, NLRP3, and IL-17 in pain and associated spinal cord alterations induced by this novel SD rat model of MOG-induced EAE, optimized to avoid confounding hindpaw-associated motor impairments/disabilities.

## Materials and methods

### Subjects

Subjects were male SD (300–325 g; Envigo) rats between 10 and 12 weeks old at arrival. Rats were housed two per cage on a 12-h light/dark cycle (lights on at 07.00 h). Experiments were conducted between 08.00 and 16.00 h. All procedures were conducted in accordance with the protocols approved by the University of Colorado Boulder Institutional Animal Care and Use Committee.

### Myelin-oligodendrocyte-glycoprotein administration

Upon arrival, the rats were randomly assigned to either the recombinant myelin-oligodendrocyte-glycoprotein_1−125_ (MOG) (VU University Medical Center, Netherlands, gifted by Dr. Anne-Marie Van Dam) or vehicle group consisting of sodium acetate (pH = 3) and incomplete Freund's adjuvant (IFA) [Sigma; St. Louis, MO; (52)]. For the initial MOG dose-effect study (Experiment 1), rats received a 100-μl intradermal injection of MOG (0, 4, 8, or 16 μg) injected at the base of the tail and were tested for motor impairments/disabilities and pain. These doses were chosen based on our previous experience conducting EAE in SD rats, in which 8 μg MOG produced deficits in fear conditioning and hippocampal glial activation in the absence of significant motor impairments/disabilities (10), as well as our experience conducting EAE in DA rats in which 16 μg MOG produced standard EAE motor impairments/disabilities that would confound behavioral testing, whereas 4 μg MOG in DA rats produced mechanical allodynia in the absence of significant motor impairments/disabilities (16). Based on these initial studies, we chose to study both 8 and 16 μg MOG doses in SD rats specifically so to avoid hindlimb motor/sensory dysfunction that could confound behavioral assessment of mechanical allodynia by the von Frey test. Importantly, whereas the 16 μg MOG dose produced mild motor impairments/disabilities that typically only involved tail paralysis that did not confound pain testing, the 8 μg MOG dose produced no motor impairments/disabilities while still allowing the expression of pain. The 8 and 16 μg MOG doses were used in Experiment 2 to test the efficacy of (+)-NTX to reverse EAE-induced pain and motor impairments/disabilities. These rats were also used for immunohistochemistry studies (i.e., Experiments 5–7), with their tissue collected on day 30 post-EAE induction [i.e., after approximately 2 weeks of (+)-NTX dosing]. In Experiments 3 and 4, which tested the ability of intrathecal delivery of NLRP3 antagonist MCC950 or an IL-17 neutralizing antibody to block EAE-induced pain, respectively, only 8 μg MOG dose was used.

### Systemic dosing by repeated acute injections of TLR2–TLR4 antagonist (+)-naltrexone

Rats in Experiment 2 received subcutaneous (+)-NTX (6 mg/kg) or equivolume saline injections three times daily (09.00, 12.00, and 15.00 h) for approximately 2 weeks during behavioral testing for a total of 18 mg/kg/day beginning on day 17 post-EAE induction. The volume of each injection administered was 1 ml/kg. This dose was chosen based on our previous studies that demonstrate this dose produces maximal antinociceptive efficacy in both models of neuropathic pain (34) and EAE (10, 16).

### Acute intrathecal injections of MCC950 and neutralizing interleukin-17 antibodies

In separate groups of rats in Experiments 3 and 4, rats were administered 8 μg MOG, and 23 (for MCC950) or 21 days later [for neutralizing interleukin-17 antibodies (anti-IL-17)], rats were administered an intrathecal injection of the NLRP3 inhibitor MCC950 (0, 0.01, 0.1, or 1 mg; Experiment 3) or anti-IL-17 (4 μg IgG control or 4 μg anti-IL-17; Experiment 4) under brief 3% isoflurane anesthesia. The 4 μg intrathecal anti-IL-17 dose was chosen based on the pilot studies (*data not shown*). The lumbar region was shaved and aseptically cleaned. An 18-gauge guide needle, with the hub removed, was inserted into the L5/L6 intervertebral space. A PE-10 catheter was inserted *via* the guide needle, pre-marked such that the proximal end of the PE-10 tubing rested over the L4–L6 lumbar spinal cord. Intrathecal injections occurred over 20 s (15 μl for MCC950 and 40 μl for anti-IL-17 followed by 2 μl of sterile saline flush) with a 30-s delay before removing the catheter and guide needle. Each rat was anesthetized for a maximum of 5 min, and none incurred observable neurological damage from the procedure.

### Behavioral tests

#### Motor scoring

All testing was conducted blind with respect to group assignment. Motor behavior was scored daily in all rats to assess the severity of their EAE symptoms. The motor score quantified physical paralysis and scoring was based on the following designations: 0 = no signs of paralysis, 1 = partial tail paralysis, 2 = full tail paralysis, 3 = hind limb weakness, 4 = partial hind limb paralysis, 5 = full hind limb paralysis, and 6 = partial upper limb paralysis. Rats that reached a score of 6 were euthanized if paralysis exceeded 1 day. Euthanized rats received a score of 7. Animals with a motor score of 3 or higher were excluded from von Frey mechanical allodynia testing to avoid hind limb motor/sensory dysfunction that could confound behavioral testing; however, importantly, all data analyses and figures presenting motor scores throughout this report include subjects that scored 3 or higher to properly assess the effects of TLR4 antagonists on this aspect of EAE.

#### Mechanical allodynia testing

All testing were conducted blind with respect to group assignment. Rats received at least three 60-min habituations to the test environment prior to behavioral testing. The von Frey test was performed as previously described in detail (53–56). Assessments were conducted approximately weekly to include testing prior to MOG administration, testing post-MOG administration to determine the level of EAE-induced pain (all experiments), and then at 12 days post (+)-NTX administration prior to tissue dissection (Experiment 2). Von Frey tests for allodynia were undertaken approximately 1 h after the second of three doses of (+)-NTX or vehicle for the day (Experiment 2). Assessments were also conducted at 3 and 24 h after intrathecal (IT) administration of the NLRP3 antagonist MCC950 and anti-IL-17 in Experiments 3 and 4, respectively. A logarithmic series of 10 calibrated Semmes-Weinstein monofilaments (von Frey hairs; Stoelting, Wood Dale, IL) were applied randomly to the left and right paws to define the threshold stimulus intensity required to elicit a paw withdrawal response. Log stiffness of the hairs ranged from manufacturer-designated 3.61 (0.40 g) to 5.18 (15.14 g) filaments. The behavioral responses were used to calculate absolute threshold (the 50% probability of response) by fitting a Gaussian integral psychometric function using a maximum-likelihood fitting method (57, 58), as described previously (56, 59). This fitting method allowed parametric analyses to be undertaken (57, 58).

### Immunohistochemistry

Then, 4 weeks after MOG administration and 12 days after continuous (+)-NTX administration, rats were deeply anesthetized with sodium pentobarbital followed by transcardial perfusion with saline (pH 7.4), followed immediately by transcardial perfusion for 5 min with 4% paraformaldehyde (pH 7.4). Lumbar spinal cords were post-fixed in 4% paraformaldehyde/0.2 M phosphate buffer (PB, pH 7.4) for 24 h and then cryo-protected in increasing concentrations (15, 20, and 30%) of sucrose in PB (pH 7.4) (Sigma-Aldrich, St. Louis, MO). Then, 5-mm representative sections of lumbar spinal cords were blocked in OCT (Fisher Scientific, Waltham, MA), frozen at −80°C, and sectioned at 16 μm on a cryostat (Leica CM1850). Sections were thaw-mounted and stored at −20°C until staining.

For rabbit ionized calcium-binding adapter molecule 1 (Iba1), rabbit glial fibrillary acidic protein (GFAP), and cluster of differentiation factor 4 (CD4) staining, slides were rinsed 3x with phosphate-buffered saline (PBS), permeabilized with 0.3% hydrogen peroxide, rinsed 3x in PBS, blocked for 1 h with 10% normal goat serum (NGS), 0.3% Triton-X in PBS, and incubated overnight at 4°C for 24 h in 2% NGS together with primary antibodies at the following dilution ratios: Iba1; 1:1,000 (019-19741; Wako, Richmond, VA), GFAP; 1:100 (Z033401-2, Dako, Carpinteria, CA), and CD4; 1:200 (ab237722; Abcam, Cambridge, MA). Slides were then rinsed 3x with PBS and incubated for 2 h in the secondary antibody at the following dilution ratio: goat anti-rabbit; 1:200 (Jackson Immuno Research, West Grove, PA). Slides were rinsed 3x in PBS, incubated in avidin-biotin complex (ABC) solution at 1:250 dilution (Vector Laboratories, Burlingame, CA) for 2 h, rinsed 3x in PBS, and incubated in inactive 3,3′-diaminobenzidine (DAB) (Sigma-Aldrich, St. Louis, MO) for 10 min. 3,3′-Diaminobenzidine was activated with B-D glucose (10 mg/ml) and slides were incubated for 8 min, rinsed 3x with PBS, and dried overnight. Slides were dehydrated in increasing concentrations of ethanol (50, 70, 95, and 100%), cleared in Citrisolv (Fisher Scientific, Waltham, MA), dried, and covered with DPX mountant (Sigma-Aldrich, St. Louis, MO). For fluoromyelin staining, slides were stained with red fluoromyelin (Thermo-Fisher Scientific, Waltham, MA) at 1:100 dilution in PBS for 20 min and then washed 3x with PBS. Slides were then coverslipped using Vectashield mountant (Vector Laboratories, Burlingame, CA).

For Iba1, GFAP, and CD4, black and white images were acquired using an Olympus BX61 microscope (Olympus America, Center Valley, PA) with Olympus Suite CellSens Dimension software. For fluoromyelin, a standard TRITC filter (excitation 558 nm) was used to visualize red-stained myelin. All images of comparison were taken using the same exposure and other acquisition settings. All images were captured at 20x magnification, except for fluoromyelin, which was captured at 10X magnification. To conduct densitometry for Iba1, GFAP, and CD4, images were converted to 32-bit, background subtracted, and then adjusted for threshold to highlight cell bodies while blinded to the treatment conditions in NIH ImageJ software. Mean fluorescent intensity for fluoromyelin was analyzed using NIH ImageJ software. Data were expressed as total area positive for staining (i.e., Iba1, GFAP, and CD4) or total fluorescent intensity (i.e., fluoromyelin) within the region of interest (i.e., dorsal lumbar spinal cord or ventral funiculus). For Iba1, GFAP, and CD4 stains, 2–4 images per animal in the region of interest were taken and analyzed individually. Due to the lack of remaining slides, for fluoromyelin stains, 2–8 pictures were taken from each animal in the analysis. For all stains, dorsal lumbar spinal cord was analyzed as this tissue is directly relevant to hindpaw mechanical allodynia (fluoromyelin data displayed no differences and are thus not shown). Finally, for CD4 and fluoromyelin stains exclusively, ventral funiculus of the spinal cord was also analyzed as a region of interest as this region composed of white matter commonly displays myelin lesions and CD4+ cell infiltration in EAE (43–45).

### Statistical analysis

Statistical analyses were conducted using GraphPad Prism v.9.31 software. Behavioral and immunohistochemistry data were analyzed by two-way ANOVA. A significant ANOVA was followed by Sidak's *Post-hoc* test to assess the differences between specific experimental groups. For all tests, statistical significance was set to *p* < 0.05.

## Results

### Experiment 1. MOG dose-effect curve for motor scores and mechanical allodynia

**Figure 1:**
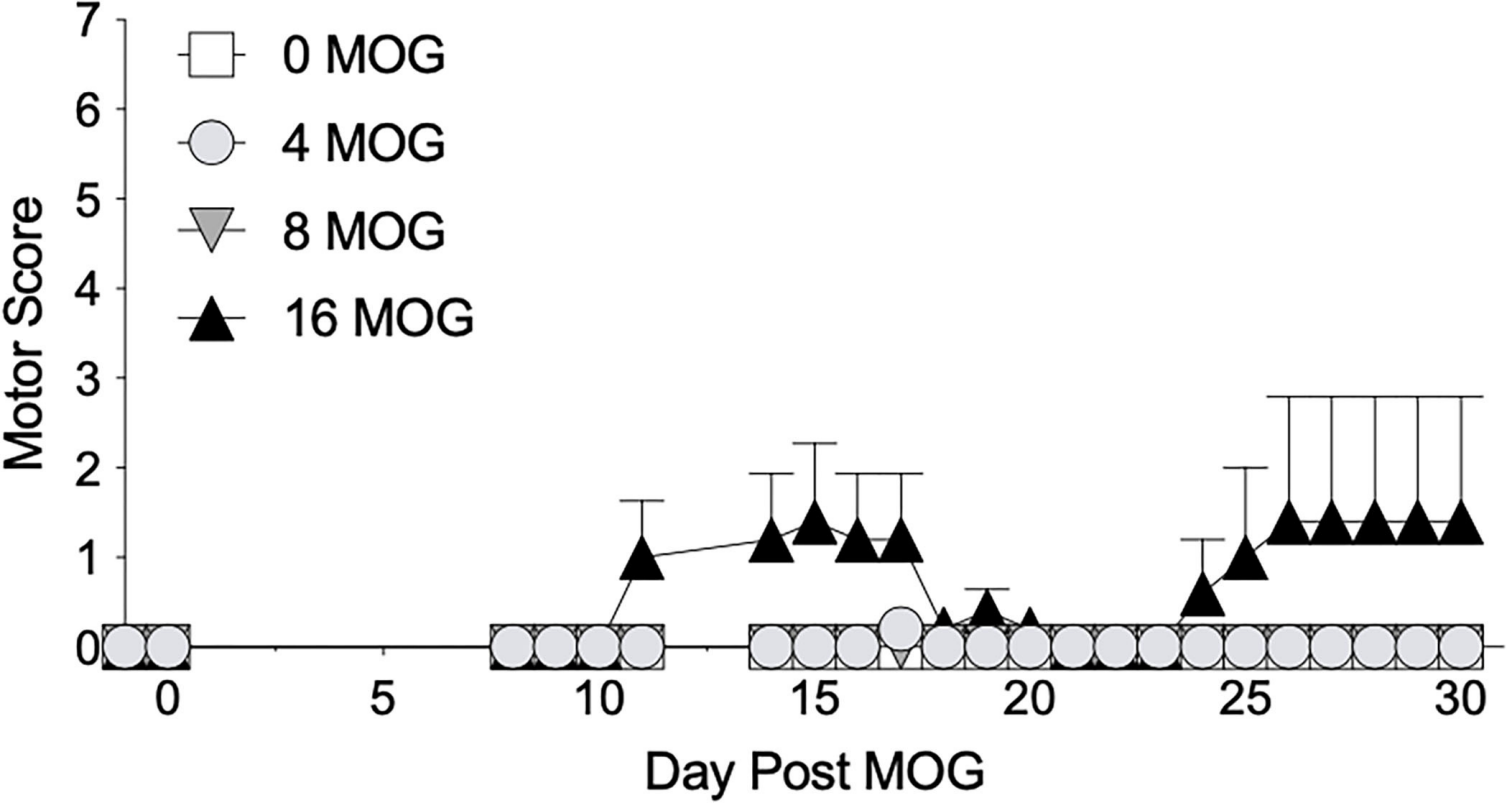
Myelin oligodendrocyte glycoprotein (MOG) in Sprague-Dawley (SD) rats produces no motor scores at low doses and mild motor scores a higher doses. Sprague-Dawley (SD) rats were baselined (BL) for motor scores followed by intradermal low-dose (0, 4, 8, and 16 μg) myelin oligodendrocyte glycoprotein (MOG). Motor scores were assessed thereafter across the timecourse shown, continuing through day 30. Neither 4 or 8 μg MOG produced motor scores, whereas 16 μg MOG produced mild motor scores. No dose of MOG reliably increased motor scores. *N* = 5/group.

We first sought to establish a dose of intradermal MOG in SD rats that would produce significant levels of long-lasting mechanical allodynia in the absence of hindlimb motor impairments/disabilities, which confounds mechanical allodynia and other behavioral testing. We thus conducted a dose-effect curve with MOG, testing doses of 0, 4, 8, and 16 μg. We found in SD rats that both 4 and 8 μg MOG doses failed to produce motor impairments/disabilities, whereas 16 μg MOG produced motor impairments/disabilities in 2 out of 5 rats (Figure 1; to see enhanced differentiation of groups with a smaller Y-axis scale, refer to Supplementary Figure S1), which ultimately led to euthanasia for one of these rats by day 19 post-MOG. No statistically reliable between-group differences in motor scores were observed across the 4-week testing period.

**Figure 2:**
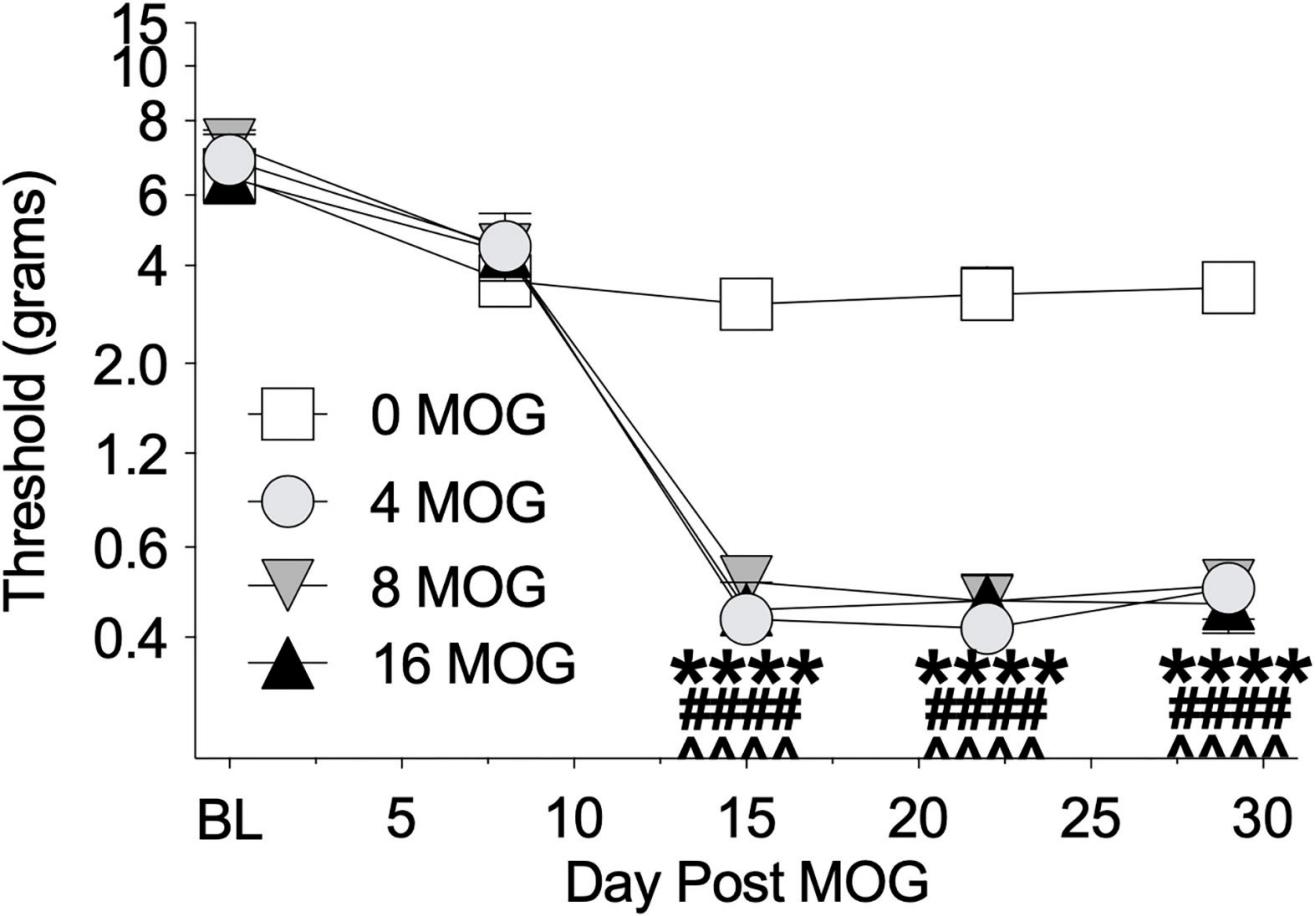
Myelin oligodendrocyte glycoprotein (MOG) in Sprague-Dawley (SD) rats produces full mechanical allodynia at all doses tested. Sprague-Dawley (SD) rats were baselined (BL) for mechanical withdrawal thresholds *via* the von Frey test, followed by intradermal low-dose (0, 4, 8, and 16 μg) myelin oligodendrocyte glycoprotein (MOG). Allodynia was assessed thereafter across the timecourse shown, continuing through day 30. All doses of MOG induced full mechanical allodynia by day 15, which lasted through day 29. Main effects of day [*F*_(4, 64)_ = 161.3; *p* < 0.0001], MOG dose [*F*_(3, 16)_ = 29.83; *p* < 0.0001], and interaction between day and MOG dose [*F*_(12, 64)_ = 10.8; *p* < 0.0001]. *N* = 5/group. *Post-hocs*: **** indicates significant differences between 0 μg MOG control and 4 μg MOG, ^*####*^ indicates significant differences between 0 μg MOG control and 8 μg MOG, ^∧∧∧∧^ indicates significant differences between 0 μg MOG control and 16 μg MOG.

We also tested these same rats in Experiment 1 before MOG dosing and then weekly thereafter for mechanical allodynia using von Frey testing (Figure 2). All doses of MOG induced full mechanical allodynia by day 15, which lasted through day 29. Sidak's *Post-hoc* test indicated that MOG significantly increased mechanical allodynia comparing 0 μg MOG and 4 μg MOG (*p* < 0.0001), 0 μg MOG and 8 μg MOG (*p* < 0.0001), and 0 μg MOG and 16 μg MOG (*p* < 0.0001) groups on days 15, 22, and 29 post-MOG.

### Experiment 2. Repeated systemic (+)-naltrexone reverses EAE-induced mechanical allodynia

**Figure 3:**
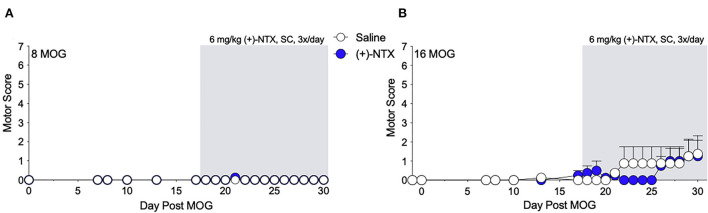
Daily systemic administration of the TLR2–TLR4 antagonist (+)-Naltrexone [(+)-NTX] does not alter EAE motor scores in Sprague-Dawley (SD) rats. Sprague-Dawley (SD) rats were baselined (BL) for motor scores followed by intradermal low-dose (0, 8, and 16 μg) myelin oligodendrocyte glycoprotein (MOG). (+)-NTX [6 mg/kg subcutaneously (SC) 3x/day] vs. saline was initiated on day 17 post-MOG, continuing through day 30 (see gray rectangle indicating span of drug delivery), and motor scores were assessed thereafter across the timecourse shown. **(A,B)** No dose of MOG reliably increased motor scores. (+)-NTX did not alter motor scores. *N* = 8/group.

As we have previously demonstrated that TLR2–TLR4 mediate mechanical allodynia in a DA EAE model (16), we next determined whether mechanical allodynia in the SD rat model was also mediated by TLR2–TLR4. Both 8 and 16 μg MOG doses were tested, as this allowed the assessment of whether (+)-NTX would be efficacious against mild motor impairments/disabilities as well as whether the presence of mild motor impairments/disabilities would alter the efficacy of (+)-NTX on mechanical allodynia in SD rats. Systemic administration of 6 mg/kg (+)-NTX 3 times/day began 17 days post-MOG administration, after the development of full mechanical allodynia. This dose was chosen based on our previous studies demonstrating the efficacy of this dosing regimen to reverse mechanical allodynia in a variety of neuropathic pain models (34–37), as well as a DA rat model of EAE (16). As in Experiment 1, 8 μg MOG did not produce motor impairments/disabilities (Figure 3A; to see enhanced differentiation of groups with a smaller Y-axis scale, refer to Supplementary Figure S2A), whereas 16 μg MOG produced motor impairments/disabilities in a subset of rats (Figure 3B; to see enhanced differentiation of groups with a smaller Y-axis scale, refer to Supplementary Figure S2B). Specifically, 4 out 8 rats developed motor impairments/disabilities in the 16 μg MOG-saline group, whereas 2 out 8 rats developed motor impairments/disabilities in the 16 μg MOG-(+)-NTX group. Moreover, one rat in the 16 μg MOG-saline group that did not develop motor impairments/disabilities died on day 22 post-MOG for unknown reasons. No statistically reliable between-group differences in motor scores were observed across the 4-week testing period.

**Figure 4:**
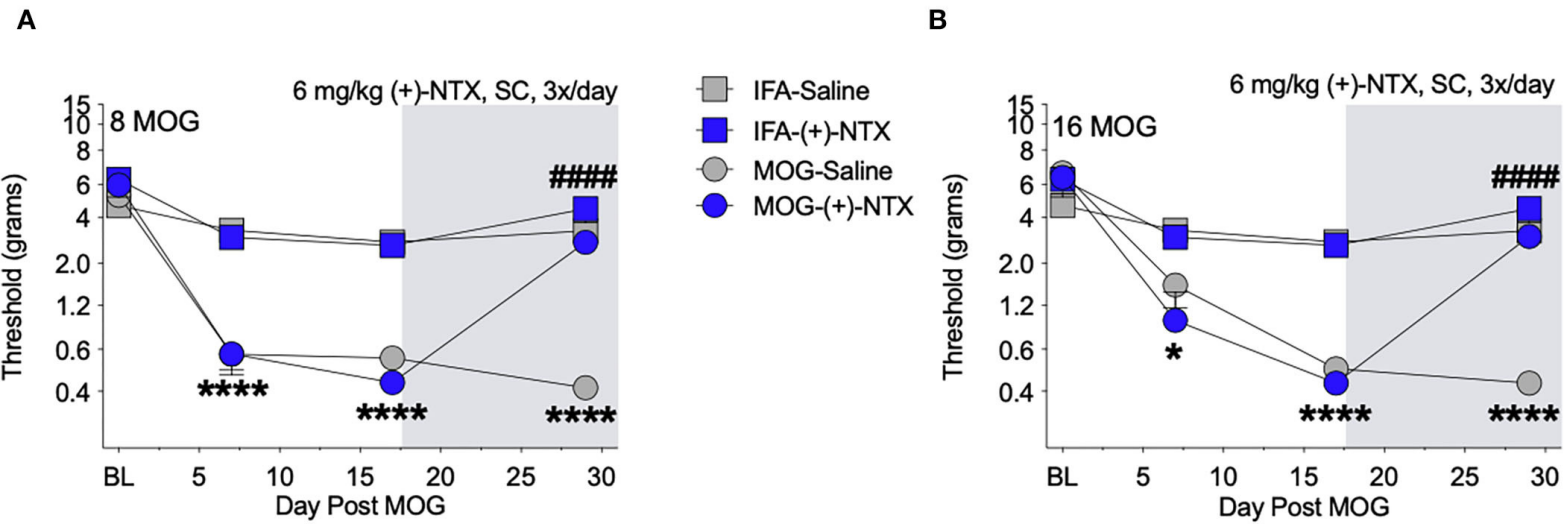
Daily systemic administration of the TLR2–TLR4 antagonist (+)-Naltrexone [(+)-NTX] reverses EAE-related pain in Sprague-Dawley (SD) rats. Sprague-Dawley (SD) rats were baselined (BL) for mechanical withdrawal thresholds *via* the von Frey test, followed by intradermal low-dose (0, 8, and 16 μg) myelin oligodendrocyte glycoprotein (MOG). (+)-NTX [6 mg/kg subcutaneously (SC) 3x/day] vs. saline was initiated on day 17 post-MOG, continuing through day 30 (see gray rectangle indicating span of drug delivery), and allodynia was assessed thereafter across the timecourse shown, continuing through day 30. All doses of MOG induced full mechanical allodynia by day 17, which lasted through day 29. **(A)** Main effects of day [*F*_(3, 84)_ = 100.80; *p* < 0.0001], treatment [*F*_(3, 28)_ = 47.51; *p* < 0.0001], and interaction between day and treatment [*F*_(9, 84)_ = 20.89; *p* < 0.0001]. **(B)** Main effects of day [*F*_(3, 72)_ = 82.07; *p* < 0.0001], [*F*_(3, 24)_ = 34.74; *p* < 0.0001], and interaction between day and treatment [*F*_(9, 72)_ = 17.73; *p* < 0.0001]. *N* = 8/group. *Post-hocs*: * indicates significant differences between 0 μg MOG-saline and each dose of MOG; ^#^ indicates significant differences between MOG-saline and MOG-(+)-NTX.

Both 8 and 16-μg doses of MOG induced full mechanical allodynia by day 17, which lasted through day 29 and was reversed by (+)-NTX administration for 8 (Figure 4A) and 16 μg (Figure 4B) MOG doses, respectively. Sidak's *Post-hoc* test indicated that MOG significantly increased mechanical allodynia between 8 and 0 μg MOG on days 7 (*p* < 0.0001), 17 (*p* < 0.0001), and 29 (*p* < 0.0001) post-MOG (Figure 4B), and between 16 and 0 μg MOG on days 7 (*p* < 0.05), 17 (*p* < 0.0001), and 29 (*p* < 0.0001) post-MOG (Figure 4B). Sidak's *Post-hoc* test also indicated that 12 days of repeated systemic (+)-NTX treatment significantly reversed MOG-induced mechanical allodynia between 8 μg MOG-saline and 8 μg MOG-(+)-NTX-treated groups (Figure 4A) and between 16 μg MOG-saline and 16 μg MOG-(+)-NTX-treated groups (Figure 4B) on day 29 post-MOG. Lumbar spinal cord tissue was collected from these groups the following day (i.e., day 30) for immunohistochemistry experiments described below.

### Experiment 3. Intrathecal administration of NLRP3 inhibitor MCCC950 reverses EAE-induced mechanical allodynia

**Figure 5:**
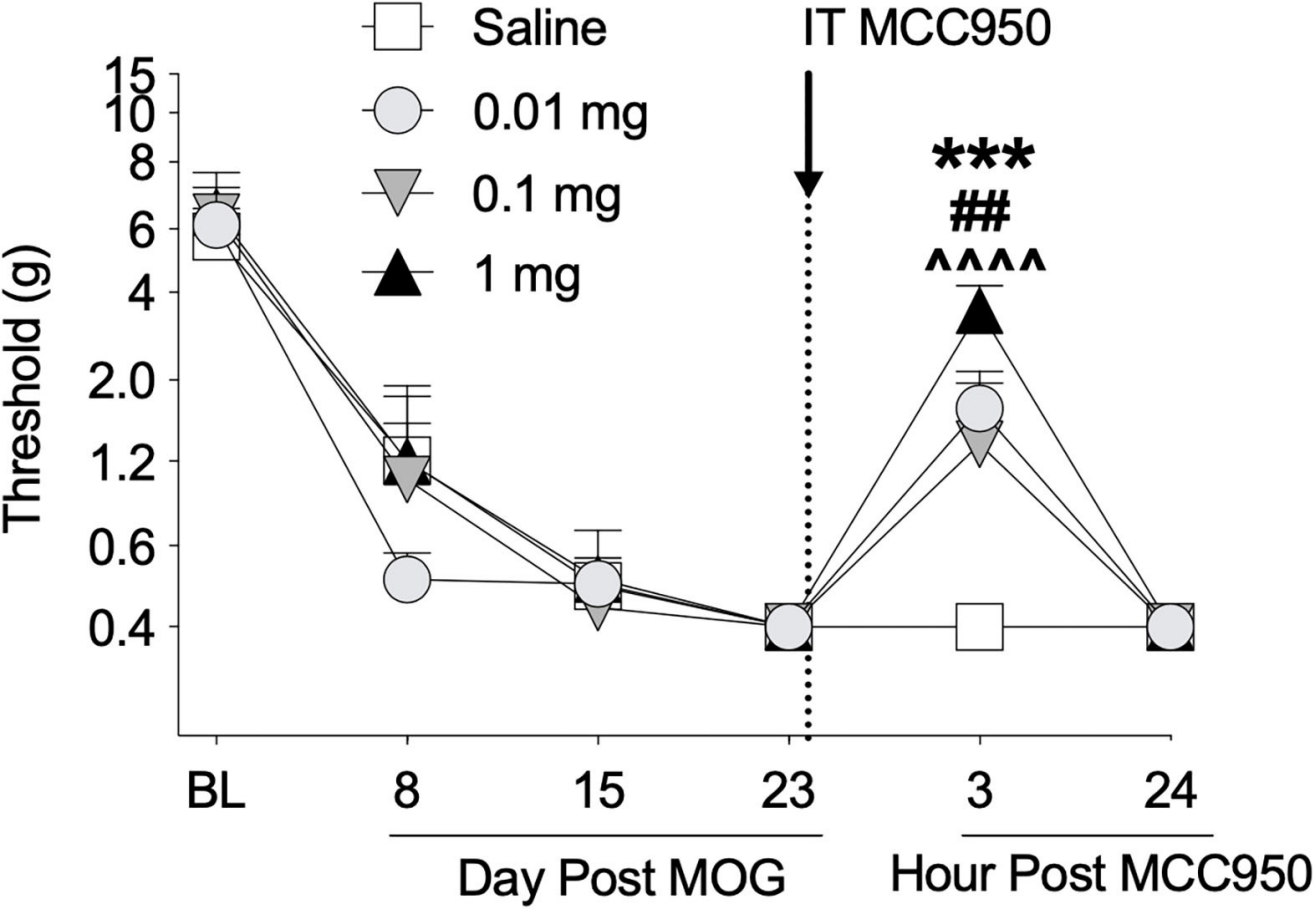
Intrathecal administration of NLRP3 antagonist MCC950 reverses EAE-related pain in Sprague-Dawley (SD) rats. Sprague-Dawley (SD) rats were baselined (BL) for mechanical withdrawal thresholds *via* the von Frey test, followed by intradermal low-dose (8 μg) myelin oligodendrocyte glycoprotein (MOG). MCC950 (0, 0.01, 0.1, 1 mg) was intrathecally administered (see dotted line/arrow indicating time of drug delivery), and allodynia was assessed prior to and at 3 and 24 h post-MCC950 delivery. Intrathecal administration of all doses of 0.01, 0.1, and 1 mg MCC950 on day 23 post-MOG significantly reversed EAE-induced mechanical allodynia at 3 h but not 24 h post-administration. Main effects of day [*F*_(5, 70)_ = 75.78; *p* < 0.01], MCC950 dose [*F*_(3, 14)_ = 4.15; *p* < 0.05], and interaction between day and MCC950 dose [*F*_(15, 70)_ = 2.83; *p* < 0.01]. *N* = 4–5/group. *Post-hocs*: *** indicates significant differences between saline control and 0.01 mg MCC950, ^*##*^ indicates significant differences between saline control and 0.1 mg, and ^∧∧∧∧^ indicates significant differences between saline control and 1 mg.

Toll-like receptors 2 and 4 agonism primes the NLRP3 inflammasome, which ultimately can lead to the production of proinflammatory cytokines such as IL-1β (63, 64) that are necessary for neuropathic pain in EAE (16, 20). Furthermore, (+)-NTX reversed EAE-induced spinal NLRP3 mRNA in our previous experiments with EAE in DA rats (16). We therefore hypothesized that spinal NLRP3 may play a functional role in EAE-induced mechanical allodynia in the SD rat EAE model presented here. In a separate group of rats, we thus intrathecally administered the NLRP3 inhibitor MCC950 on day 23 post-8 μg MOG administration and assessed rats for mechanical allodynia using von Frey testing. As in Experiments 1 and 2, 8 μg MOG induced full mechanical allodynia by day 15 post-MOG, which remained stable through day 23 post-MOG (Figure 5); two-way ANOVA comparing all days before MCC950 administration in all treatment groups [i.e., days baseline (BL), 8, 15, and 23] indicated a significant effect of day [*F*_(3, 42)_ = 97.37; *p* < 0.0001]. Sidak's *Post-hoc* test indicated significant differences within the saline group between BL and Days 8 (*p* < 0.0001), 15 (*p* < 0.0001), and 23 (*p* <0.0001), within the 0.01 mg group between BL and Days 8 (*p* < 0.0001), 15 (*p* < 0.0001), and 23 (*p* < 0.0001), within the 0.1 mg group between BL and Days 8 (*p* < 0.0001), 15 (*p* < 0.0001), and 21 (*p* < 0.0001) and within the 1 mg group between BL and Days 8 (*p* < 0.00001), 15 (*p* < 0.0001), and 23 (*p* < 0.0001). Intrathecal administration of all doses of 0.01, 0.1, and 1 mg MCC950 on day 23 post-MOG significantly reversed EAE-induced mechanical allodynia at 3 h but not 24 h post-administration (Figure 5). Sidak's *post-hoc* test indicated significant differences between saline and 0.01 (*p* < 0.001), 0.1 (*p* < 0.01), and 1 mg (*p* < 0.0001) MCC950 groups at 3 h, but not 24 h post-intrathecal MCC950 administration (Figure 5).

### Experiment 4. Intrathecal administration of interleukin-17 neutralizing antibodies reverses EAE-induced mechanical allodynia

**Figure 6:**
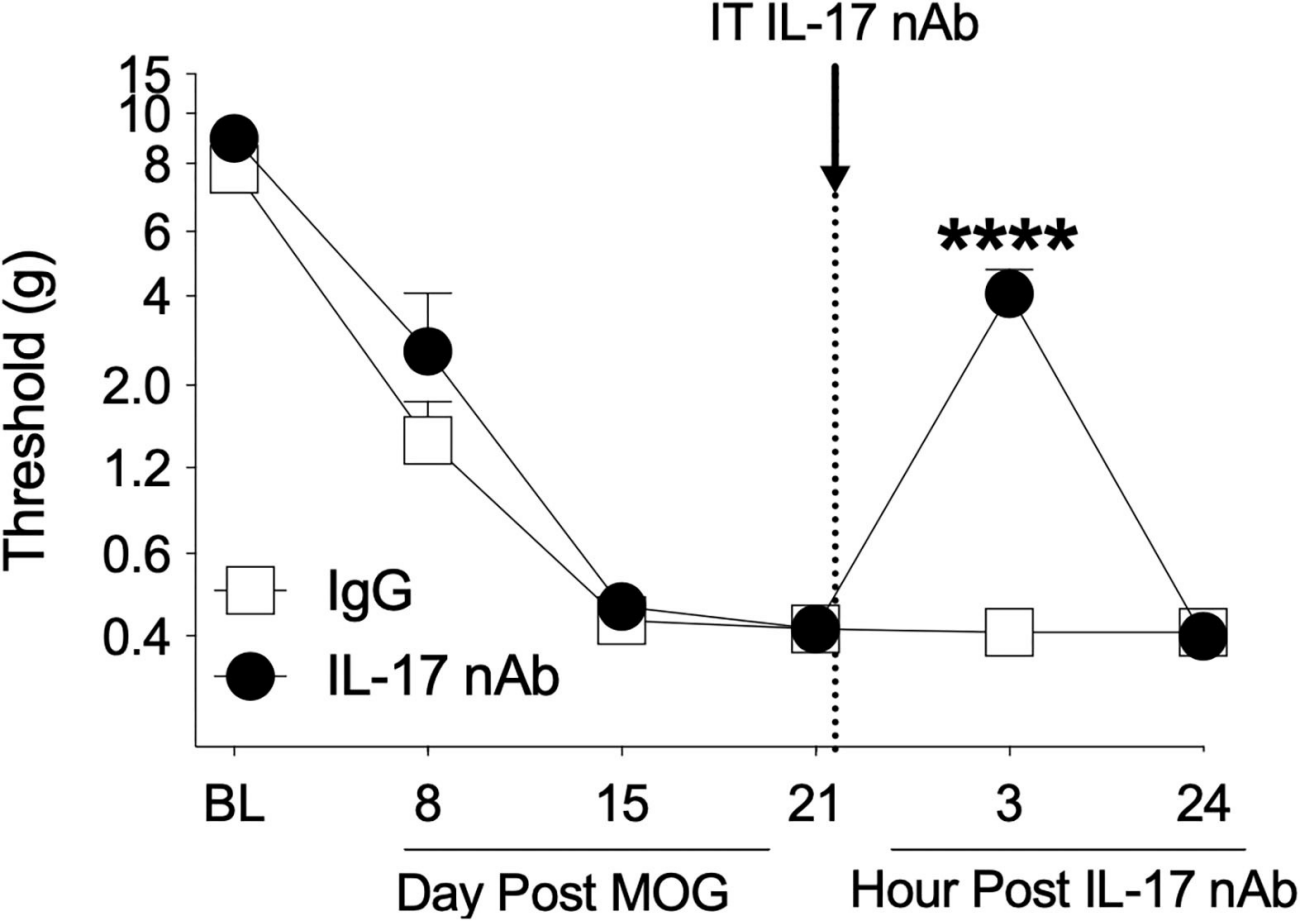
Intrathecal administration of interleukin-17 neutralizing antibodies (IL-17 nAb) reverses EAE-related pain in Sprague-Dawley (SD) rats. Sprague-Dawley (SD) rats were baselined (BL) for mechanical withdrawal thresholds *via* the von Frey test, followed by intradermal low-dose (8 μg) myelin oligodendrocyte glycoprotein (MOG). Approximately 4 μg of Interleukin-17 neutralizing antibodies (IL-17 nAb) or IgG control was intrathecally administered (see dotted line/arrow indicating time of drug delivery), and allodynia was assessed prior to and at 3 and 24 h post-IL-17 nAb delivery. Intrathecal administration of IL-17 nAb reversed EAE-induced mechanical allodynia at 3 h but not 24 h post-administration. Main effects of day [*F*_(5, 70)_ = 142.5; *p* < 0.0001], IL-17 nAb [*F*_(1, 14)_ = 19.57; *p* < 0.001], and interaction between day and IL-17 nAb [*F*_(5, 70)_ = 17.48; *p* < 0.001]. *N* = 8/group. *Post-hocs*: **** indicates significant differences between saline control and each dose of IL-17 nAb.

The Th17 cell cytokine IL-17 has been shown to necessary for pain in models of peripheral nerve injury (60, 66–68) and EAE (32), as well to EAE (23, 40, 69, 70) and MS disease expression (71). Therefore, we assessed whether spinal IL-17 contributes to the mechanical allodynia in the SD rat EAE model presented here. To test this hypothesis, we intrathecally administered IL-17 neutralizing antibody on day 21 post-8 μg MOG administration and assessed rats for mechanical allodynia using von Frey testing in a separate group of rats. As in Experiments 1–3, 8 μg MOG induced full mechanical allodynia by day 15 post-MOG, which remained stable through day 21 post-MOG (Figure 6); two-way ANOVA comparing timepoints before IL-17 neutralizing antibody administration in all treatment groups (i.e., days BL, 8, 15, and 21) indicated a significant effect of day [*F*_(1, 21)_ = 148.2; *p* < 0.0001]. Sidak's *Post-hoc* test indicated significant differences within the IgG group between BL and days 8 (*p* < 0.001), 15 (*p* < 0.0001), and 21 (*p* < 0.0001) and within the IL-17 group between BL and days 8 (*p* < 0.05), 15 (*p* < 0.0001), and 21 (*p* < 0.0001). Intrathecal administration of 4 μg IL-17 neutralizing antibody or 4 μg IgG control on day 21 post-MOG significantly reversed EAE-induced mechanical allodynia at 3 h but not 24 post-administration (Figure 6). Sidak's *Post-hoc* test indicated significant differences between IgG control and IL-17 neutralizing antibody at 3 h but not 24 h post-intrathecal IL-17 neutralizing antibody administration (*p* < 0.0001) (Figure 6).

### Experiment 5. Repeated systemic (+)-Naltrexone reverses EAE-induced dorsal horn lumbar spinal cord glial immunoreactivity

**Figure 7:**
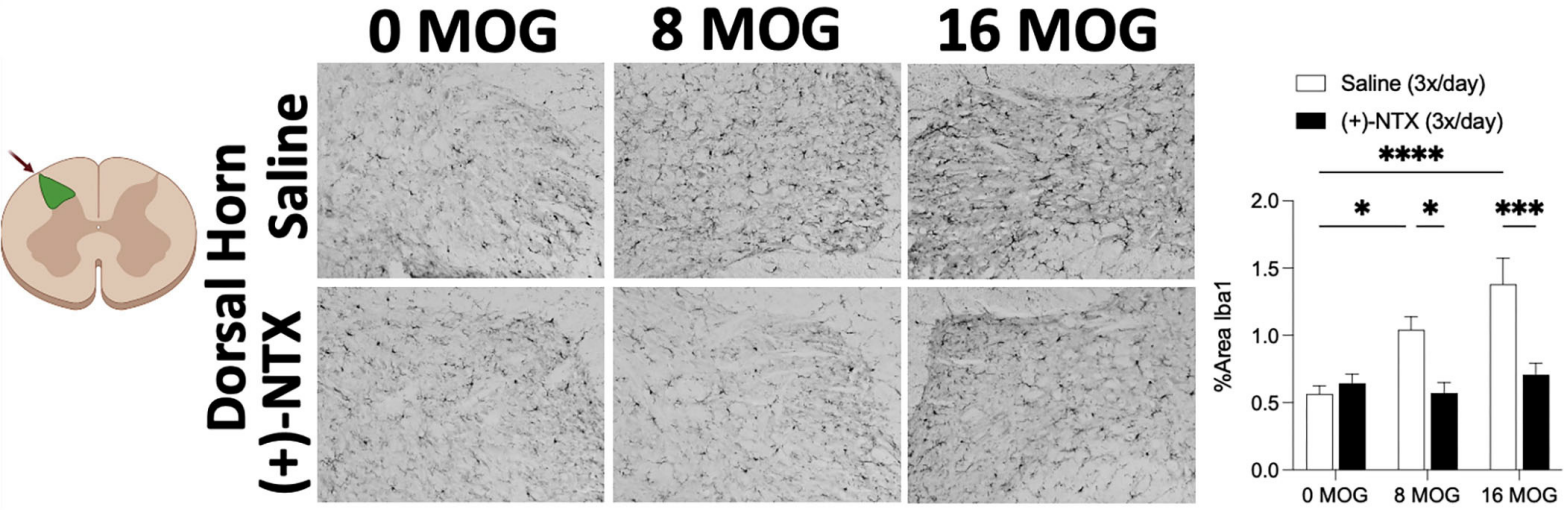
Myelin oligodendrocyte glycoprotein (MOG) induces lumbar spinal cord dorsal horn Iba1 immunoreactivity that is reversed by daily systemic administration of the TLR2–TLR4 antagonist (+)-Naltrexone [(+)-NTX]. Sprague-Dawley (SD) from Experiment 2 received intradermal low-dose (0, 8, and 16 μg) myelin oligodendrocyte glycoprotein (MOG). Spinal cord tissue was collected on day 30 post-MOG administration and during 13 days of daily (+)-NTX administration. Both doses of MOG increased expression of the microglial immunoreactivity marker Iba1 in dorsal horn of lumbar spinal cord, which was reversed to 0 μg MOG control levels by (+)-NTX administration. Main effects of MOG [*F*_(2, 86)_ = 9.66; *p* < 0.001], (+)-NTX [*F*_(2, 86)_ = 19.57; *p* < 0.0001], and interaction between MOG and (+)-NTX [*F*_(2, 86)_ = 7.81; *p* < 0.001]. *N* = 3–8/group, with 2–4 pictures analyzed per subject. *Post-hocs*: * indicates significant differences between groups (*p < 0.05, **p < 0.01, ***p < 0.001, ****p < 0.0001).

**Figure 8:**
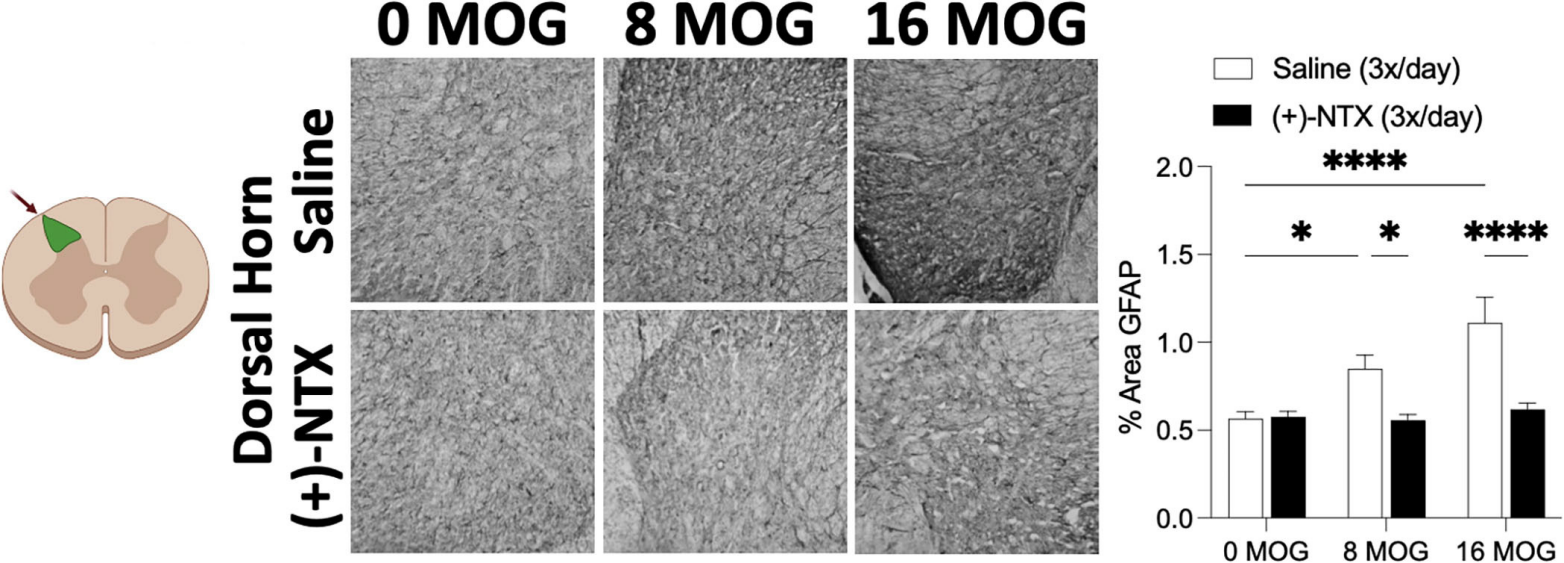
Myelin oligodendrocyte glycoprotein (MOG) induces lumbar spinal cord dorsal horn GFAP immunoreactivity that is reversed by daily systemic administration of the TLR2–TLR4 antagonist (+)-Naltrexone [(+)-NTX]. Sprague-Dawley (SD) from Experiment 2 received intradermal low-dose (0, 8, and 16 μg) myelin oligodendrocyte glycoprotein (MOG). Spinal cord tissue was collected on day 30 post-MOG administration and during 13 days of daily (+)-NTX administration. Both doses of MOG increased expression of the astrocyte immunoreactivity marker GFAP in dorsal horn of lumbar spinal cord, which was reversed to 0 μg MOG control levels by (+)-NTX administration. Main effects of MOG [*F*_(2,80)_ = 10.02; *p* = 0.0001], (+)-NTX [*F*_(1,80)_ = 24.89; *p* < 0.05], and interaction between MOG and (+)-NTX [*F*_(2,80)_ = 7.79; *p* < 0.001]. *N* = 5–8/group with 2–4 pictures analyzed per subject. *Post-hocs*: * indicates significant differences between groups (*p < 0.05, ****p < 0.0001).

Spinal cord glial activation has been shown to be necessary for neuropathic pain (29), and we have previously shown that spinal dorsal horn glial immunoreactivity in a DA rat EAE model is correlated with EAE-induced mechanical allodynia, both of which are reversed by TLR2–TLR4 blockade with daily systemic administration of (+)-NTX (16). We thus hypothesized that EAE in SD rats would induce increased spinal glial immunoreactivity that would also be blocked by daily (+)-NTX administration. Rats from Experiment 2 were used for these studies, with their tissue collected on day 30 post-MOG administration after the final behavioral tests and 13 days of consecutive daily (+)-NTX dosing. Both doses of MOG increased expression of the microglial immunoreactivity marker Iba1 (Figure 7 for 20X pictures and Supplementary Figure S3 for 40X pictures) and astrocyte immunoreactivity marker GFAP (Figure 8 for 20X pictures and Supplementary Figure S4 for 40X pictures) in dorsal horn of lumbar spinal cord, which were both reversed to 0 μg MOG control levels by (+)-NTX administration. For Iba1, Sidak's *Post-hoc* test indicated significant differences between 0 μg MOG-saline and 8 μg MOG-saline (*p* < 0.05), 0 μg MOG-saline and 16 μg MOG-saline (*p* < 0.0001), 8 μg MOG-saline and 8 μg MOG-(+)-NTX (*p* < 0.05), and 8 μg MOG-saline and 8 μg MOG-(+)-NTX (*p* < 0.001). For GFAP, Sidak's *post-hoc* test indicated significant differences between 0 μg MOG-saline and 8 μg MOG-saline (*p* < 0.05), 0 μg MOG-saline and 16 μg MOG-saline (*p* < 0.0001), 8 μg MOG-saline and 8 μg MOG-(+)-NTX (*p* < 0.05), and 8 μg MOG-saline and 8 μg MOG-(+)-NTX (*p* < 0.0001).

### Experiment 6. Neither EAE or repeated systemic (+)-naltrexone alter lumbar dorsal horn or ventral funiculus CD4+ T cell immunoreactivity

**Figure 9:**
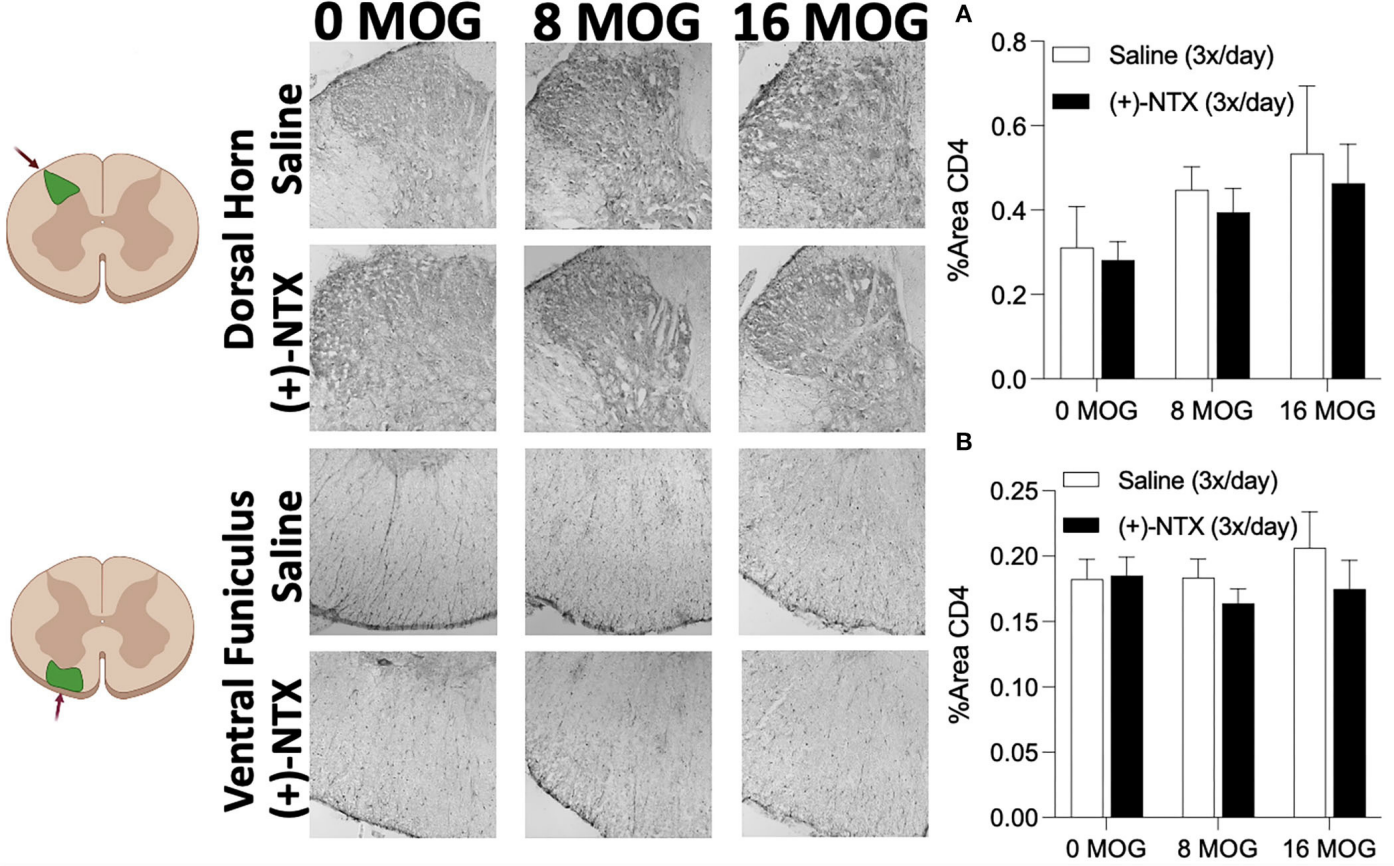
Myelin oligodendrocyte glycoprotein (MOG) does not induce lumbar spinal cord dorsal horn or ventral funiculus CD4 immunoreactivity. Sprague-Dawley (SD) from Experiment 2 received intradermal low-dose (0, 8, and 16 μg) myelin oligodendrocyte glycoprotein (MOG). Spinal cord tissue was collected on day 30 post-MOG administration and during 13 days of daily (+)-NTX administration. Neither dose of MOG increased expression of the T cell immunoreactivity marker CD4 in either dorsal horn **(A)** or ventral funiculus **(B)** of lumbar spinal cord. (+)-NTX administration also did not alter CD4 immunoreactivity in either region. *N* = 5–8/group with 2–4 pictures analyzed per subject.

As we demonstrated in Experiment 4 that spinal Th17 cell cytokine IL-17 is necessary for EAE-induced mechanical allodynia in this SD rat model (Figure 5), we hypothesized that EAE may induce increased CD4+ cell infiltration of lumbar dorsal horn and that (+)-NTX might block this effect. Using tissue from rats in Experiment 2, however, we determined that neither MOG or (+)-NTX produced significant differences in CD4 immunoreactivity in lumbar dorsal horn. A two-way ANOVA indicated a trend for MOG to increase CD4 immunoreactivity in this region that did not reach a level statistical significance (Figure 9A for 20X pictures; *p* = 0.06, refer to Supplementary Figure S5A for 40X pictures).

We next investigated CD4 immunoreactivity in lumbar ventral funiculus. We hypothesized that ventral funiculus would not display increased CD4 immunoreactivity due to the lack of motor impairments/disabilities present in the model, as CD4+ cell infiltration in white matter is commonly associated with motor impairments/disabilities and demyelinating lesions (43–45). As predicted, we did not find any significant group differences in CD4 immunoreactivity in this region (Figure 9B for 20X pictures, refer to Supplementary Figure S5B for 40X pictures).

### Experiment 7. Repeated systemic (+)-naltrexone does not alter EAE-induced lumbar ventral funiculus lumbar spinal cord demyelination

**Figure 10:**
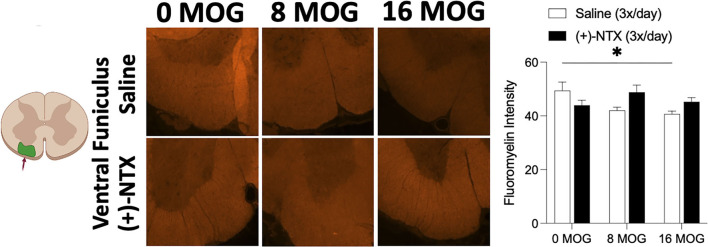
Myelin oligodendrocyte glycoprotein (MOG) induces lumbar spinal ventral funiculus demyelination. Sprague-Dawley (SD) from Experiment 2 received intradermal low-dose (0, 8, and 16 μg) myelin oligodendrocyte glycoprotein (MOG). Spinal cord tissue was collected on day 30 post-MOG administration and during 13 days of daily (+)-NTX administration. Only the 16-μg dose of MOG significantly decreased fluoromyelin staining intensity of ventral funiculus of lumbar spinal cord; however, this was not significantly reversed by (+)-NTX administration. Significant interaction between MOG and (+)-NTX treatment [*F*_(2,151)_ = 5.89; *p* < 0.01]. *N* = 5–8/group with 2–8 pictures analyzed per subject. *Post-hocs*: * indicates significant differences between groups (*p < 0.05).

As demyelination is a critical component of EAE and MS (6, 74), we wanted to explore in this SD rat model whether MOG would induce spinal cord demyelination detectable by immunohistochemistry, and whether this demyelination would be reversed by TLR2–TLR4 blockade. Fluoromyelin fluorescently stains myelin; hence, a decrease in fluorescence indicates the areas of demyelination (65, 75). This is an assessment of the presence of demyelination using fluoromyelin fluorescent intensity of lumbar ventral funiculus from tissue collected from rats in Experiment 2. Myelin-oligodendrocyte-glycoprotein produced a significant decrease in fluoromyelin fluorescent intensity (Figure 10). Sidak's *Post-hoc* test indicated a significant difference between 0 and 16 μg MOG (*p* < 0.05); however, this decreased fluoromyelin staining intensity was not reversed by (+)-NTX despite a trend to increase fluoromyelin fluorescent intensity after both 8 and 16 μg MOG doses (Figure 10).

Finally, as few subjects in this SD rat model displayed significant motor impairments/disabilities of 4 or above (i.e., only 3 out 16 subjects), we conducted a qualitative analysis for demyelination and CD4 immunoreactivity in lumbar ventral funiculus of two rats from the 16 μg saline group that displayed opposing severities of motor impairments/disabilities but equal levels of mechanical allodynia throughout the study (Supplementary Figures S6A,B). Notably, the subject that displayed higher motor impairments/disabilities could not be tested for mechanical allodynia on the final day (i.e., day 29) due to motor impairments/disabilities causing hindlimb impairment (the absence of datapoint marked by arrow on Supplementary Figure S6). Interestingly, the rat that displayed partial bilateral hindlimb paralysis at the time of dissection had more extensive demyelination (Supplementary Figure S6D) and CD4 immunoreactivity (Supplementary Figure S6F) than in the rat that displayed no motor impairments/disabilities at the time of dissection and which only expressed partial tail paralysis on a single day prior to dissection (Supplementary Figures S6C,E for demyelination and CD4 immunoreactivity, respectively). We could not conduct quantitative analysis or assess the ability of (+)-NTX to reverse more extensive demyelination or CD4 immunoreactivity from this tissue due to the lack of subjects that displayed these phenomena.

## Discussion

The goal of these studies was to characterize a mild MOG-induced SD rat model of EAE that was optimized for the study of longer timecourses of EAE-induced pain in the absence of confounding hindpaw-related motor impairments/disabilities. Our data show that (1) MOG at all doses tested (4, 8, and 16 μg) produced full mechanical allodynia by 2 weeks post-MOG, with no motor impairments/disabilities produced at the lower doses and mostly mild motor impairments/disabilities at the highest dose, (2) the TLR2–TLR4 antagonist (+)-NTX reverses EAE-induced mechanical allodynia without affecting motor impairments/disabilities while also reversing increased microglial and astrocyte immunoreactivity of dorsal horn of lumbar spinal cord, (3) both spinal NLRP3 and IL-17 are necessary for EAE-induced pain in this model, (4) EAE induced demyelination of ventral funiculus at the highest dose tested, which was not reversed by the 2-week (+)-NTX treatment, and (5) CD4+ T cell infiltration of dorsal horn or ventral funiculus of lumbar spinal cord was not reliably increased after MOG administration, but was correlated in one individual subjects that did display demyelination and severe motor impairments/disabilities.

Modeling pain-related behavior as well as other complex behaviors in EAE generally requires one of three strategies to avoid hindpaw-related motor impairments/disabilities that confound behavioral testing. The first strategy involves testing subjects prior to the development of motor impairments/disabilities. Various studies have employed this strategy and reported detectable pain within the first week following EAE induction (15, 20, 22, 23). While this strategy has been proven to be very useful to understand the mechanisms of EAE-induced pain and the efficacy of treatments to treat such pain, this strategy typically is restricted to the assessments only within the first week following EAE induction, before the development of hindpaw-related motor impairments/disabilities that confound behavioral testing. Contrastingly, a second strategy is to reduce the dose of antigen, adjuvant, and/or blood brain barrier disrupting agent (i.e., pertussis toxin) delivered to the subjects, which allows for testing of pain-behavior over a more extended timecourse. Using this strategy, subjects do not develop severe motor impairments/disabilities that confound behavioral testing or require euthanasia. As with the first strategy, several studies have reported extended pain-related behavior in EAE using this low-dose approach (18, 19, 21), including recent studies from our laboratory in which male and female DA rats in a low-dose MOG-induced EAE model displayed pain for at least 2 months following EAE induction (16). Importantly, many patients with MS report experiencing pain for years prior to the development of motor impairments/disabilities and official diagnosis of MS (62). This finding, in conjunction with findings that EAE causes pain prior to motor disturbance onset (15, 20, 22, 23), suggests that low-dose EAE may selectively model early MS-associated pain, implicating the importance in assessing low-dose models to develop a greater understanding of MS-related pain treatments and mechanisms. Finally, a final strategy to avoid hindpaw-related motor impairments/disabilities in EAE is to choose a strain of subject and antigen combination that does not induce severe EAE. The most commonly used strains of rats used for MOG-induced EAE studies are DA, Lewis, or Brown Norway rats due to their susceptibly to develop reliable levels of motor impairments/disabilities following MOG administration (76). Strains such as SD rats are less commonly used to study motor impairments/disabilities in EAE. In particular, standard EAE studies in SD rats to date have used spinal cord homogenate to generate an autoimmune response against myelin, which does produce reliable and severe motor impairments/disabilities at standard doses (77, 78). In contrast, by employing both the second and third strategies, we have recently reported increased memory deficits in the absence of significant motor impairments/disabilities in a low-dose MOG-induced SD rat model of EAE (10). Here, we extend these findings to the characterization of similar low-dose MOG-induced SD rat model of EAE optimized for the study of EAE-induced pain.

We found that the TLR2–TLR4 antagonist (+)-NTX reverses EAE-induced pain and increased glial immunoreactivity in the dorsal horn of lumbar spinal cord in the SD rat model presented here. These findings are consistent with previous studies using the models of peripheral and centrally-induced neuropathic pain (34–37), as well as in EAE pain models (16). Regarding EAE specifically, we have shown that pain in a low-dose MOG-induced EAE model in DA rats is also reversed by TLR2–TLR4 antagonist treatment in both male and female rats using a similar timecourse and dosing treatment regimen for both MOG and (+)-NTX (16). In the DA rat model, however, sex differences were discovered in which increased astrocyte immunoreactivity of dorsal horn of lumbar spinal cord only occurred in females but not males, whereas increased microglial immunoreactivity was shown in both sexes (16). Contrastingly, in the SD rat model presented here, both microglial and astrocyte immunoreactivities were increased in the lumbar dorsal horn of male rats. While it is possible that increased astrocyte immunoreactivity in male DA rats requires higher MOG doses, it is important to note that motor impairments/disabilities were already present in the DA rat model at a dose of 4 μg MOG, whereas motor impairments/disabilities were only present in the SD rat model at the highest dose of MOG we tested (i.e., 16 μg). These data suggest that the threshold for inducing increased astrocyte immunoreactivity appears to be higher in male DA rats than in male SD rats, as well as in female DA rats. Sex differences were not explored in the SD rat model, as the explicit goal here was to compare the results with prior studies, which by-and-large used males as subjects. Expanding the SD rat model to females would be a valuable topic of future research. Sex differences are recognized as an important area of study in all research, including in clinical MS-related pain, as female patients generally express MS-related pain more frequently than in male patients (79). These data highlight the importance of developing various models of EAE that can involve different mediators and cell types to assess the efficacy of treatments and explore new mechanisms of MS-associated pain.

In the CNS, TLR2 and TLR4 are primarily expressed on astrocytes and microglia (29, 38), but are also present on neurons (38, 39) and CNS infiltrating T cells (40, 41). Toll-like receptor 2 and 4 are activated by DAMPs from breakdown of myelin and other damaged and inflamed cells, including products such as heat shock protein 60 (HSP60), HSP70, high mobility group box 1 (HMGB1), alpha-synuclein, hyaluronic acid, fibrinogen, and biglycan (61, 73, 80–86). Ligation of TLR2 and/or TLR4 then induces NF-kB activation that primes the NLRP3 inflammasome and can ultimately lead to the production of the proinflammatory cytokine IL-1β that has been shown in various rodent models to cause pain upon intrathecal dosing (29, 30). Moreover, we have previously reported that spinal IL-1β is necessary for EAE-induced pain in male DA rats (16), whereas here we show that the NLRP3 inflammasome is necessary for SD rat EAE-induced pain (Figure 5). Consistent with these findings, we have shown previously that daily treatment with (+)-NTX reverses pain that is correlated with normalizing spinal NLRP3 and IL-1β mRNA in male DA rats (16), and we have also reported that this same treatment regimen reverses memory deficits in both DA and SD male rat models of low-dose EAE, which is similarly associated with normalizing EAE-induced increased hippocampal glial activation, and NLRP3, IL-1β, and IL-17 mRNAs in male DA rats (10). While we did not explicitly test whether spinal IL-1β is necessary for pain in the SD rat model due to the lack of continued funding, given the necessity of NLRP3 for pain in this model and the strong relationship between NLRP3 activation and IL-1β release, IL-1β would be predicted to also be necessary for pain in the SD rat model presented here. Whether sex differences exist in the SD rat model for these measures remains to be defined. Finally, it has previously been reported that both IL-1β and NLRP3 are necessary for EAE-induced motor disturbance in mice (72, 87), which is thought to function through activation of Th17 cells *via* IL-1β and granulocyte-macrophage colony-stimulating factor (GM-CSF) signaling (88). Future studies should also investigate whether these pathways may be necessary for EAE-induced motor impairments/disabilities in SD rats using spinal cord homogenate as an antigen to induce more severe motor impairments/disabilities, as the MOG-induced SD male rat model presented here is not well-suited to address this question.

As CD4+ Th17 T cells also express TLR2 (41) and TLR4 (40) and the primary proinflammatory cytokine IL-17 that is released from Th17 cells has recently been shown to contribute to EAE-induced pain in mice (32), we chose to investigate whether IL-17 was necessary for EAE pain in the SD rat EAE model. We also examined whether reduced CD4+ cell immunoreactivity in the dorsal horn of lumbar spinal cord correlated with decreased pain after (+)-NTX treatment. We found that while spinal IL-17 was necessary for EAE-induced pain in SD rats, neither EAE nor (+)-NTX treatment altered lumbar dorsal horn levels of CD4 immunoreactivity. There are several possible reasons for the discrepancy between these findings. One possible reason is that sufficiently few CD4+ cells infiltrate the spinal cord in this low-dose EAE model that they avoid detection by immunohistochemistry. Contrastingly, employing flow cytometry allows the assessment of small subsets of diverse populations of T cells (89). In support of this hypothesis, we found that the highest dose of MOG tested in the SD rat model (i.e., 16 μg) produced a statistical trend to increase CD4 immunoreactivity that nearly reached a level of statistical significance (i.e., *p* = 0.06; Figure 9). It is thus possible that flow cytometry would be able to more sensitively detect the changes in these cell populations after mild EAE induction. Adding flow cytometry to future studies would likely be of value, as the mild nature of the low-dose EAE model would be expected to not produce as much CD4+ cell infiltration as in standard EAE models. A second reason we may not have detected significant increases in CD4 immunoreactivity is that many T cells in EAE and MS do not cross the blood brain barrier and are instead compartmentalized in meningeal and/or perivascular locations (44). Due to the limitations in the methods of spinal cord dissections used for immunohistochemistry in these studies, the meningeal and perivascular regions did not remain intact, and thus, it cannot be ruled out that a significant population of CD4+ cells were affected by either EAE and/or (+)-NTX treatment in these locations. Future studies will need to be designed to assay meningeal/perivascular populations of cells in the SD rat model. It is also possible that the timepoint chosen for immunohistochemistry (i.e., 1 month post-MOG administration) was not early enough to observe the changes in CD4+ cell infiltrates, as T cell infiltration often occurs alongside the development of motor impairments/disabilities that typically begin approximately 2 weeks after MOG administration (43–45), including in a standard EAE study in SD rats that used spinal cord homogenate as the antigen (78). Finally, CD8+ T cells are known to be present in higher numbers of demyelinating lesions in both EAE and patients with MS than CD4+ T cells. However, while some studies have shown that CD8+ cells can release IL-17 under some conditions, Th17 cells are considered the primary cells that produce IL-17 (42, 90). It remains to be determined whether CD8 immunoreactivity would have been increased to a greater degree than CD4 immunoreactivity in the SD rat EAE model presented here. Finally, TLR2 and TLR4 on CD4+ cells have been shown to be necessary for EAE-induced motor impairments/disabilities in mice (40, 41), and thus, they should also be investigated with appropriate standard EAE SD rat models (i.e., using spinal cord homogenate as an antigen) to determine whether they are also necessary for motor impairments/disabilities in SD rats.

Finally, we found that the highest dose of MOG tested in the SD rat model (i.e., 16 μg) produced a significant decrease in fluoromyelin intensity in ventral funiculus of lumbar spinal cord (Figure 10), indicating that demyelination of white matter was ongoing at this dose. However, the 2-week (+)-NTX treatment only produced a trend to reverse this effect, which did not reach a level statistical significance (Figure 10). It is unknown whether a longer course of treatment may have proven effective. Furthermore, qualitative analysis in Supplementary Figure S6 shows that a single rat with severe motor impairments/disabilities was correlated with a robust decrease in fluoromyelin intensity of ventral funiculus, and that this decreased fluoromyelin intensity highly correlated with increased CD4 immunoreactivity in this same rat. A second rat that received 16 μg MOG but did not display severe motor impairments/disabilities was included for comparison and was not found to show such changes in fluoromyelin intensity or CD4 immunoreactivity, despite both animals expressing full mechanical allodynia throughout the study (Supplementary Figure S6). These data suggest that immunohistochemistry of a representative section of lumbar spinal cord likely cannot account for the differences in spatial location of demyelinating lesions that occur in a low-dose EAE model, such that individual subject data for myelin staining intensity and CD4 cell infiltration have the potential to be greatly skewed depending on whether the section contains a lesion or not. Moreover, as few subjects displayed severe motor impairments/disabilities in the 16 μg MOG group, it is also quite likely that fewer and less severe demyelinating lesions were produced compared to standard EAE models. This unfortunately made it difficult to assess the effects of (+)-NTX on these phenomena. As the goal of this study was to produce a low-dose SD model of EAE to study pain, future studies will be required to address these questions. Our data here are consistent with previous studies demonstrating that more severe motor impairments/disabilities that are associated with more severe demyelination and inflammatory cell infiltration (91, 92).

In conclusion, this study provides the first characterization of a low-dose MOG-induced SD rat EAE model optimized for the study of pain, so as to induce stable allodynia across many weeks. We found that doses of 8 and 16 μg MOG could produce long-lasting mechanical allodynia in the absence of motor impairments/disabilities in male SD rats, which was associated with increased spinal glial activation and demyelination. Furthermore, we demonstrated that both spinal NLRP3 and IL-17 were necessary for MOG-induced mechanical allodynia in this model. Finally, we showed that 2 weeks of systemic administration of the TLR2–TLR4 antagonist (+)-NTX reversed MOG-induced mechanical allodynia and decreased spinal glial activation without affecting spinal T cell expression or demyelination. As up to 92% of patients with MS experience pain (1), including many patients who report pain for many years prior to MS diagnosis (62), these findings highlight the importance of utilizing such models to explore the mechanisms and treatments that can improve pain and thus the overall quality of life of patients with MS.

## Data availability statement

The original contributions presented in the study are included in the article/Supplementary materials, further inquiries can be directed to the corresponding author/s.

## Ethics statement

The animal study was reviewed and approved by CU-Boulder IACUC.

## Author contributions

AK: conceptualization, methodology, validation, formal analysis, investigation, writing—original draft, writing—review and editing, project administration, and funding acquisition. MC, KH, and EM: investigation, and writing—review and editing. TL, SL, BW, LT, and AS: investigation. A-MVD and KR: resources. SM: resources writing—review and editing, and funding acquisition. LW: resources, conceptualization, writing—review and editing, project administration, and funding acquisition.

## Funding

This work was supported in part by the grants from the National Institute of Neurological Disorders and Stroke (R01NS097313), the University of Colorado Biological Sciences Initiative, and by the Intramural Research programs of the National Institute on Drug Abuse and National Institute on Alcohol Abuse and Alcoholism, National Institute on Health.

## Conflict of interest

The authors declare that the research was conducted in the absence of any commercial or financial relationships that could be construed as a potential conflict of interest.

## Publisher's note

All claims expressed in this article are solely those of the authors and do not necessarily represent those of their affiliated organizations, or those of the publisher, the editors and the reviewers. Any product that may be evaluated in this article, or claim that may be made by its manufacturer, is not guaranteed or endorsed by the publisher.

## Supplementary material

Supplementary Figure S1 Myelin oligodendrocyte glycoprotein (MOG) in Sprague-Dawley (SD) rats produces no motor scores at low doses and mild motor scores a higher doses. Figure 1 is re-represented here as Supplementary Figure S2 with a smaller Y-axis scale for enhanced differentiation of groups.

Supplementary Figure S2 Daily systemic administration of the TLR2–TLR4 antagonist (+)-Naltrexone [(+)-NTX] does not alter EAE motor scores in Sprague-Dawley (SD) rats. Figure 3 is re-represented here as Supplementary Figure S3 with a smaller Y-axis scale for enhanced differentiation of groups.

Supplementary Figure S3 Myelin oligodendrocyte glycoprotein (MOG) induces lumbar spinal cord dorsal horn Iba1 immunoreactivity that is reversed by daily systemic administration of the TLR2–TLR4 antagonist (+)-Naltrexone [(+)-NTX]. Figure 7 Iba1 immunohistochemistry pictures are re-represented here in 40X magnification for enhanced differentiation of cell staining.

Supplementary Figure S4 Myelin oligodendrocyte glycoprotein (MOG) induces lumbar spinal cord dorsal horn GFAP immunoreactivity that is reversed by daily systemic administration of the TLR2–TLR4 antagonist (+)-Naltrexone [(+)-NTX]. Figure 8 GFAP immunohistochemistry pictures are re-represented here in 40X magnification for enhanced differentiation of cell staining.

Supplementary Figure S5 Myelin oligodendrocyte glycoprotein (MOG) induces lumbar spinal cord dorsal horn GFAP immunoreactivity that is reversed by daily systemic administration of the TLR2–TLR4 antagonist (+)-Naltrexone [(+)-NTX]. Figure 9 CD4 immunohistochemistry pictures are re-represented here in 40X magnification for enhanced differentiation of cell staining.

Supplementary Figure S6 Qualitative analysis of individual rat demyelination and CD4 immunoreactivity in ventral funiculus correlates with motor scores, but not mechanical allodynia. Two individual rats from the 16 μg myelin oligodendrocyte glycoprotein (MOG)-saline control group in Experiment 2 displayed equal levels of mechanical allodynia while displaying opposing levels of motor impairments/disabilities throughout the course of the study (Supplementary Figure S1A vs. Supplementary Figure S1B). The rat that displayed higher motor impairments/disabilities could not be tested for mechanical allodynia on the final day (i.e., day 29) due to motor impairments/disabilities causing hindlimb impairment (absence of datapoint marked by arrow on Supplementary Figure S1). The rat with higher motor scores **(B)** displayed decreased myelin staining intensity **(D)** and increased CD4 immunoreactivity **(F)** compared to the rat with low motor scores **(A,C,E)**. No quantitative analysis of these data were conducted due to the lack of subjects that displayed the phenomena depicted in panels **B,D,F**.
