# Supplementary figures and images for: Involvement of TLR2–TLR4, NLRP3, and IL-17 in pain induced by a novel Sprague-Dawley rat model of experimental autoimmune encephalomyelitis
Source: Front Pain Res (Lausanne). 2022 Sep 13;3:932530. doi: 10.3389/fpain.2022.932530 (PMC9513159; doi:10.3389/fpain.2022.932530)

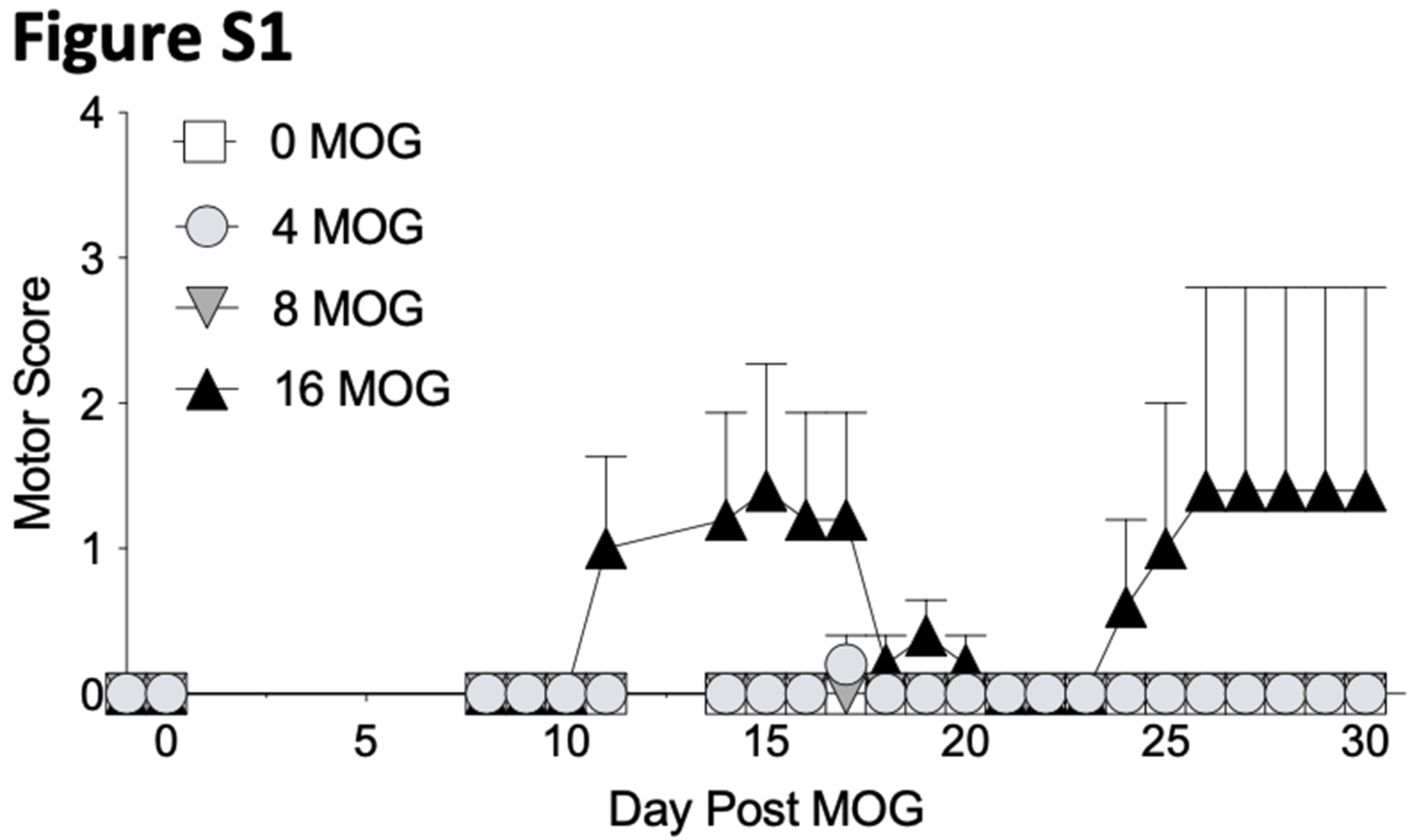

Supplement: Supplementary Figure S1 — Myelin oligodendrocyte glycoprotein (MOG) in Sprague-Dawley (SD) rats produces no motor scores at low doses and mild motor scores a higher doses. Figure 1 is re-represented here as Supplementary Figure S2 with a smaller Y-axis scale for enhanced differentiation of groups. [file Image_1.tiff]

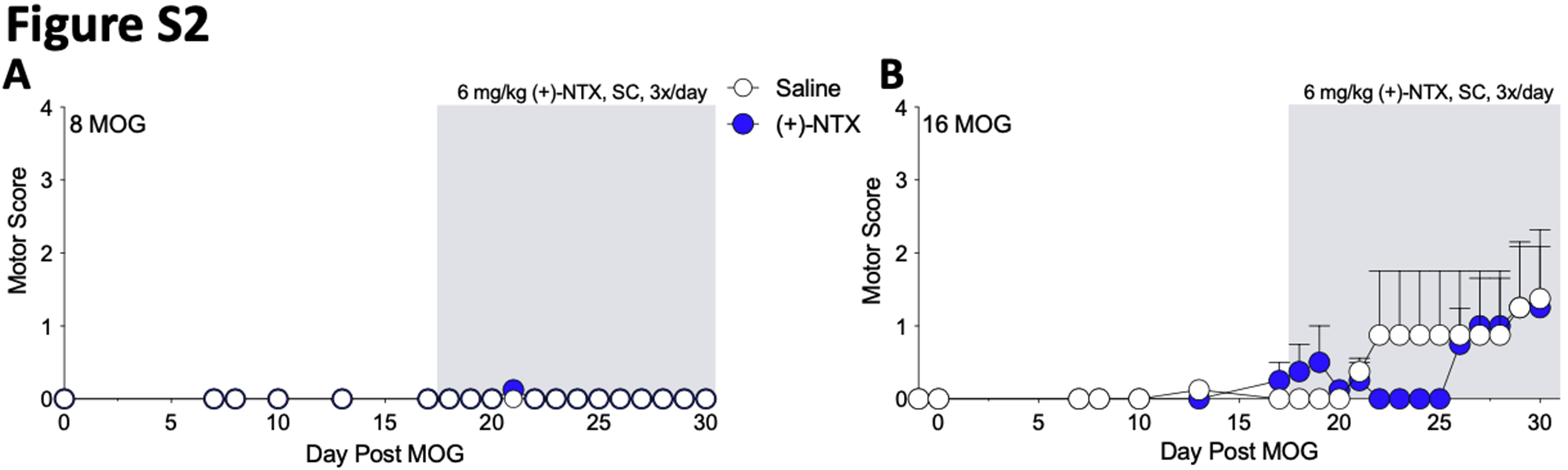

Supplement: Supplementary Figure S2 — Daily systemic administration of the TLR2–TLR4 antagonist (+)-Naltrexone [(+)-NTX] does not alter EAE motor scores in Sprague-Dawley (SD) rats. Figure 3 is re-represented here as Supplementary Figure S3 with a smaller Y-axis scale for enhanced differentiation of groups. [file Image_2.tiff]

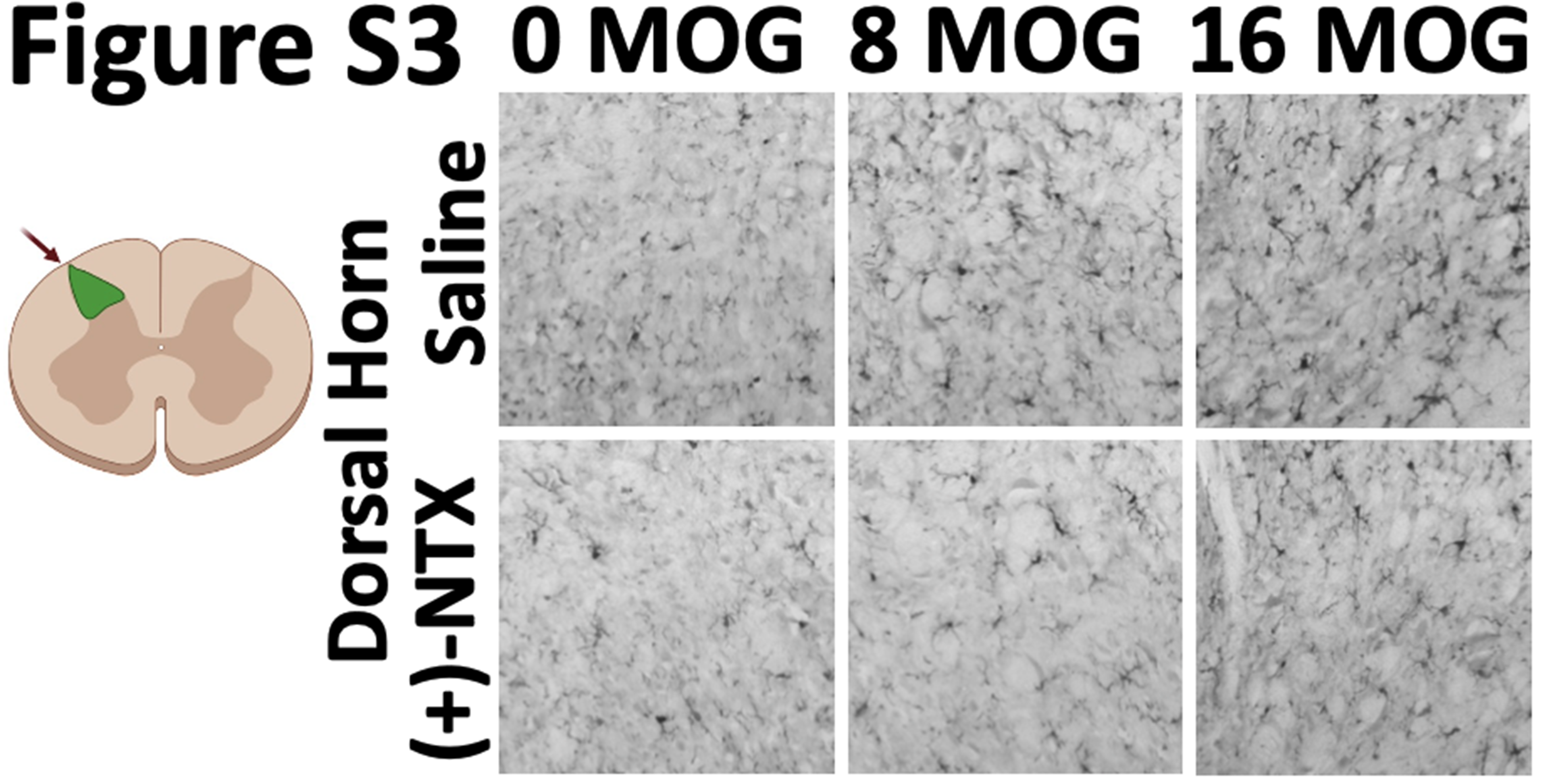

Supplement: Supplementary Figure S3 — Myelin oligodendrocyte glycoprotein (MOG) induces lumbar spinal cord dorsal horn Iba1 immunoreactivity that is reversed by daily systemic administration of the TLR2–TLR4 antagonist (+)-Naltrexone [(+)-NTX]. Figure 7 Iba1 immunohistochemistry pictures are re-represented here in 40X magnification for enhanced differentiation of cell staining. [file Image_3.tiff]

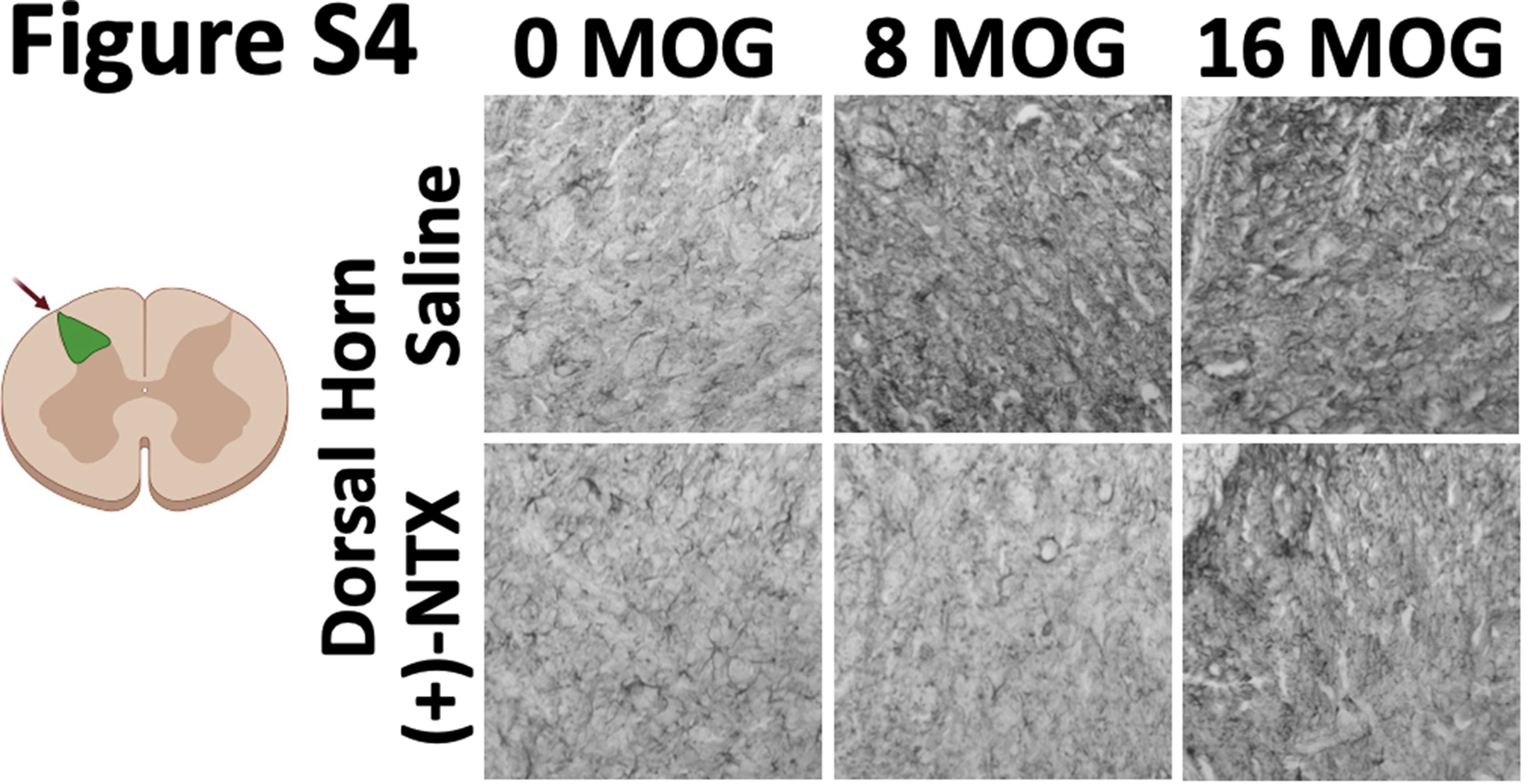

Supplement: Supplementary Figure S4 — Myelin oligodendrocyte glycoprotein (MOG) induces lumbar spinal cord dorsal horn GFAP immunoreactivity that is reversed by daily systemic administration of the TLR2–TLR4 antagonist (+)-Naltrexone [(+)-NTX]. Figure 8 GFAP immunohistochemistry pictures are re-represented here in 40X magnification for enhanced differentiation of cell staining. [file Image_4.tiff]

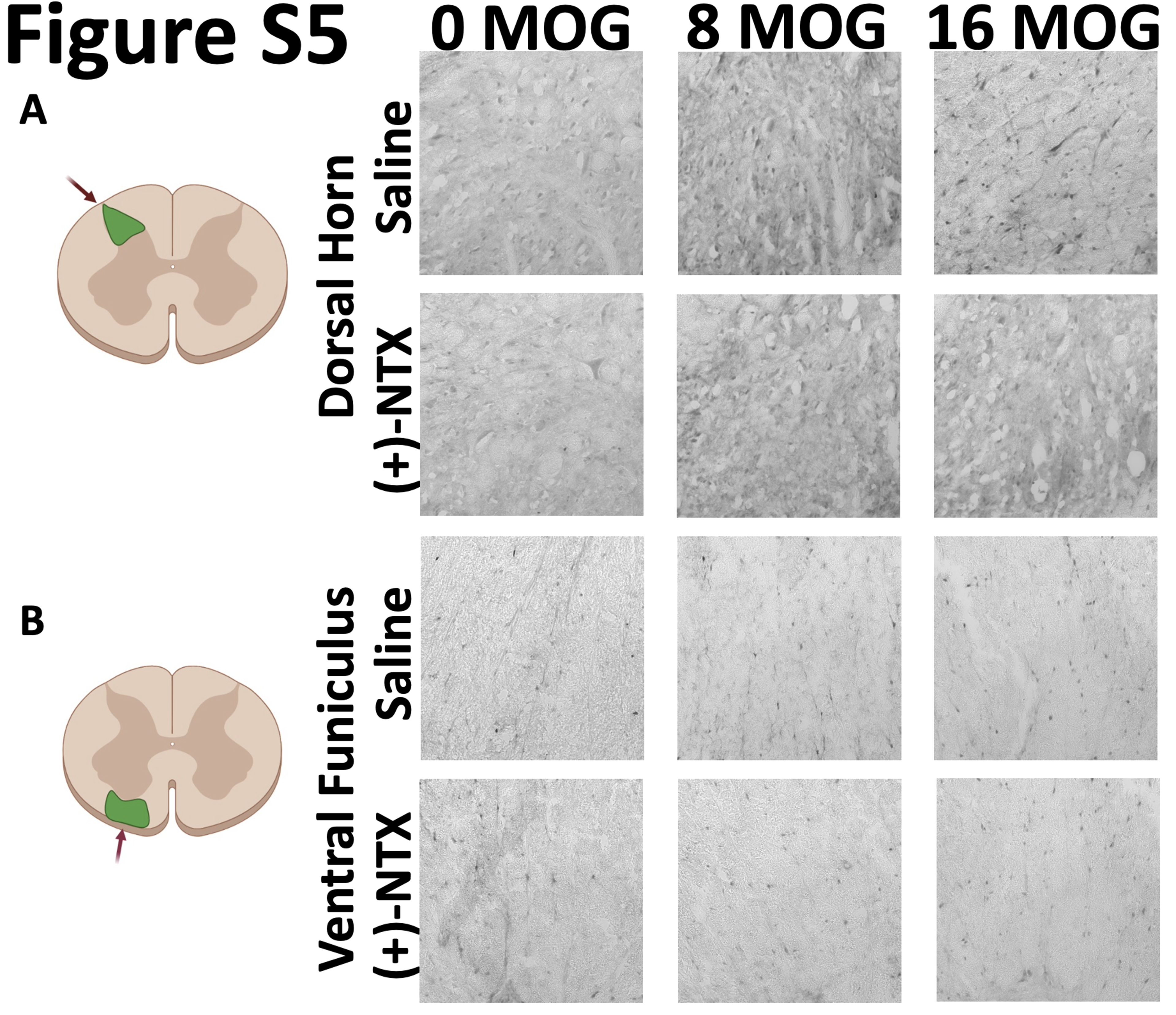

Supplement: Supplementary Figure S5 — Myelin oligodendrocyte glycoprotein (MOG) induces lumbar spinal cord dorsal horn GFAP immunoreactivity that is reversed by daily systemic administration of the TLR2–TLR4 antagonist (+)-Naltrexone [(+)-NTX]. Figure 9 CD4 immunohistochemistry pictures are re-represented here in 40X magnification for enhanced differentiation of cell staining. [file Image_5.tiff]

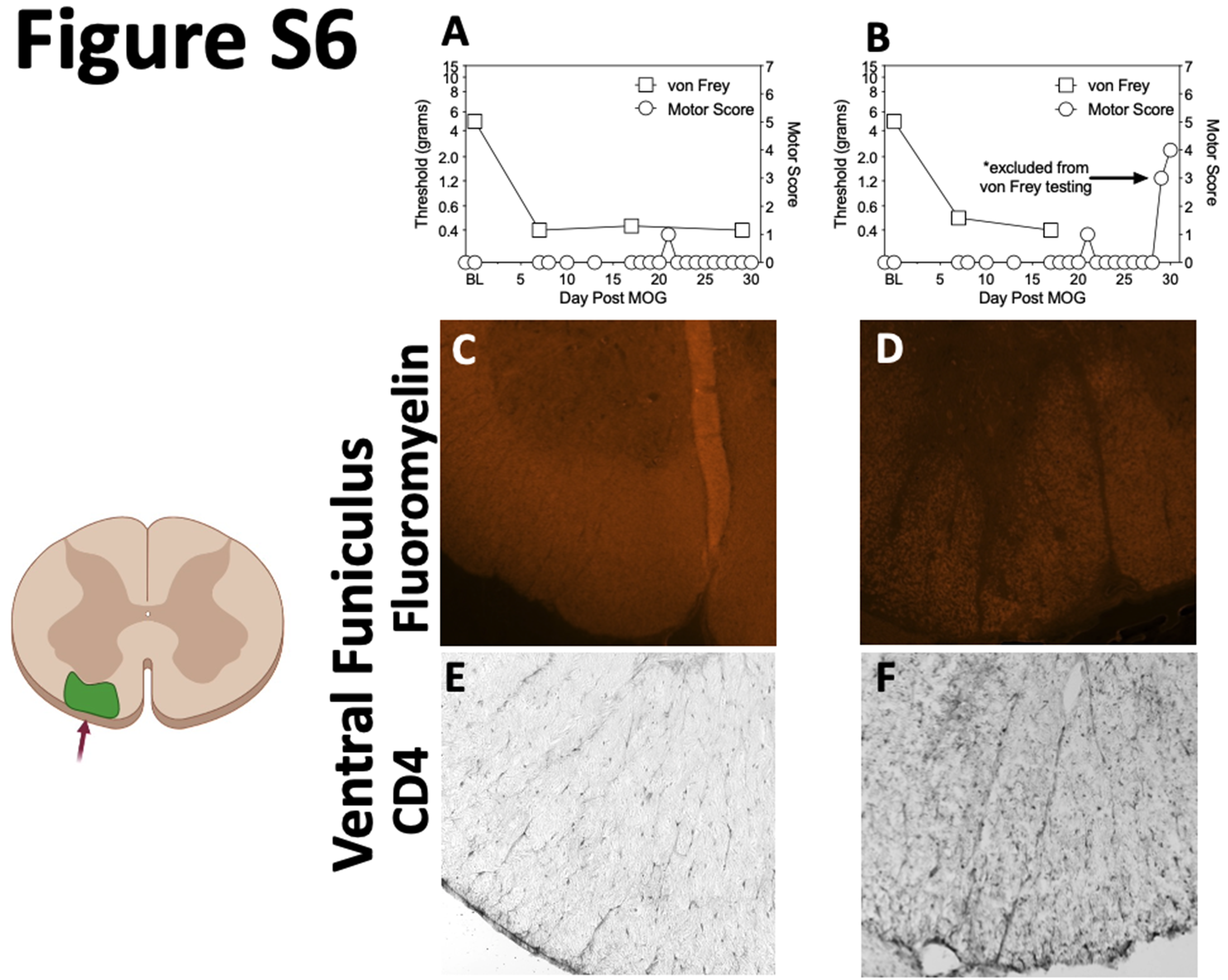

Supplement: Supplementary Figure S6 — Qualitative analysis of individual rat demyelination and CD4 immunoreactivity in ventral funiculus correlates with motor scores, but not mechanical allodynia. Two individual rats from the 16 μg myelin oligodendrocyte glycoprotein (MOG)-saline control group in Experiment 2 displayed equal levels of mechanical allodynia while displaying opposing levels of motor impairments/disabilities throughout the course of the study (Supplementary Figure S1A vs. Supplementary Figure S1B). The rat that displayed higher motor impairments/disabilities could not be tested for mechanical allodynia on the final day (i.e., day 29) due to motor impairments/disabilities causing hindlimb impairment (absence of datapoint marked by arrow on Supplementary Figure S1). The rat with higher motor scores (B) displayed decreased myelin staining intensity (D) and increased CD4 immunoreactivity (F) compared to the rat with low motor scores (A,C,E). No quantitative analysis of these data were conducted due to the lack of subjects that displayed the phenomena depicted in panels B,D,F. [file Image_6.tiff]
